# Author Correction: Whole-genome sequencing of 1,171 elderly admixed individuals from Brazil

**DOI:** 10.1038/s41467-022-29575-z

**Published:** 2022-03-30

**Authors:** Michel S. Naslavsky, Marilia O. Scliar, Guilherme L. Yamamoto, Jaqueline Yu Ting Wang, Stepanka Zverinova, Tatiana Karp, Kelly Nunes, José Ricardo Magliocco Ceroni, Diego Lima de Carvalho, Carlos Eduardo da Silva Simões, Daniel Bozoklian, Ricardo Nonaka, Nayane dos Santos Brito Silva, Andreia da Silva Souza, Heloísa de Souza Andrade, Marília Rodrigues Silva Passos, Camila Ferreira Bannwart Castro, Celso T. Mendes-Junior, Rafael L. V. Mercuri, Thiago L. A. Miller, Jose Leonel Buzzo, Fernanda O. Rego, Nathalia M. Araújo, Wagner C. S. Magalhães, Regina Célia Mingroni-Netto, Victor Borda, Heinner Guio, Carlos P. Rojas, Cesar Sanchez, Omar Caceres, Michael Dean, Mauricio L. Barreto, Maria Fernanda Lima-Costa, Bernardo L. Horta, Eduardo Tarazona-Santos, Diogo Meyer, Pedro A. F. Galante, Victor Guryev, Erick C. Castelli, Yeda A. O. Duarte, Maria Rita Passos-Bueno, Mayana Zatz

**Affiliations:** 1 Human Genome and Stem Cell Research Center, University of São Paulo, São Paulo, SP Brazil; 2 Department of Genetics and Evolutionary Biology, Biosciences Institute, University of São Paulo, São Paulo, SP Brazil; 3 Hospital Israelita Albert Einstein, São Paulo, SP Brazil; 4 Instituto da Criança, Faculdade de Medicina da Universidade de São Paulo, São Paulo, SP Brazil; 5 Orthopedic Research Labs, Boston Children’s Hospital and Department of Genetics, Harvard Medical School, Boston, MA USA; 6 Laboratório DASA, São Paulo, Brazil; 7 Laboratory of Genome Structure and Ageing, European Research Institute for the Biology of Ageing, University Medical Center Groningen, Groningen, Netherlands; 8 São Paulo State University (UNESP), Molecular Genetics and Bioinformatics Laboratory, School of Medicine, Botucatu, State of São Paulo Brazil; 9 São Paulo State University (UNESP), Department of Pathology, School of Medicine, Botucatu, State of São Paulo Brazil; 10 Departamento de Química, Faculdade de Filosofia, Ciências e Letras de Ribeirão Preto, Universidade de São Paulo, Ribeirão Preto, São Paulo Brazil; 11 Centro de Oncologia Molecular, Hospital Sirio-Libanes, São Paulo, Brazil; 12 Department of Biochemistry, Institute of Chemistry, University of São Paulo São Paulo, São Paulo, Brazil; 13 Bioinformatics Graduate program, University of São Paulo, São Paulo, Brazil; 14 Departamento de Genética, Ecologia e Evolução, Instituto de Ciências Biológicas, Universidade Federal de Minas Gerais, Belo Horizonte, MG Brazil; 15 Núcleo de Ensino e Pesquisa, Instituto Mário Penna, Belo Horizonte, MG Brazil; 16 Laboratorio de Biotecnologia y Biologia Molecular, Instituto Nacional de Salud, Lima, Peru; 17 Universidad de Huánuco, Huánuco, Peru; 18 Division of Cancer Epidemiology and Genetics, National Cancer Institute, Bethesda, MD USA; 19 Instituto de Saúde Coletiva, Universidade Federal da Bahia, Salvador, BA 40110-040 Brazil; 20 Center for Data and Knowledge Integration for Health, Institute Gonçalo Muniz, Fundação Oswaldo Cruz, Salvador, BA Brazil; 21 Instituto de Pesquisas René Rachou, Fundação Oswaldo Cruz, Belo Horizonte, MG Brazil; 22 Programa De Pós-Graduação em Saúde Pública, Universidade Federal de Minas Gerais, Belo Horizonte, MG Brazil; 23 Programa de Pós-Graduação em Epidemiologia, Universidade Federal de Pelotas, Pelotas, RS Brazil; 24 Mosaico Translational Genomics Initiative, Universidade Federal de Minas Gerais, Belo Horizonte, MG 31270-901 Brazil; 25 Facultad de Salud Pública y Administración, Universidad Peruana Cayetano Heredia, Lima, Peru; 26 Instituto de Estudos Avançados Transdisciplinares, Universidade Federal de Minas Gerais, Belo Horizonte, MG 31270-901 Brazil; 27 Medical-Surgical Nursing Department, School of Nursing, University of São Paulo, São Paulo, SP Brazil; 28 Epidemiology Department, Public Health School, University of São Paulo, São Paulo, SP Brazil

**Keywords:** Mobile elements, Haplotypes, Data publication and archiving, Structural variation, Rare variants

Correction to: *Nature Communications* 10.1038/s41467-022-28648-3, published online 04 March 2022.

The original version of this Article contained an error in the title, which was previously incorrectly given as ‘Whole-genome sequencing of 1,171 elderly admixed individuals from São Paulo, Brazil’. The correct version removes the word “São Paulo”. This has been corrected in both the PDF and HTML versions of the Article.

