# Supplementary material for: Whole-genome sequencing of 1,171 elderly admixed individuals from Brazil
Source: Nat Commun. 2022 Mar 4;13:1004. doi: 10.1038/s41467-022-28648-3 (PMC8897431; doi:10.1038/s41467-022-28648-3)
Supplement: Supplementary file 1 — Supplementary Information [file 41467_2022_28648_MOESM1_ESM.pdf]

Supplementary Information

Whole-genome sequencing of 1,171 elderly admixed individuals from São Paulo, Brazil

Michel S. Naslavsky*[1,2,3†], Marilia O. Scliar*[1], Guilherme L. Yamamoto*[1,4,5,6], Jaqueline Yu Ting Wang[1], Stepanka Zverinova[7], Tatiana Karp[7], Kelly Nunes[2], José Ricardo Magliocco Ceroni[1], Diego Lima de Carvalho[1], Carlos Eduardo da Silva Simões[1], Daniel Bozoklian[1], Ricardo Nonaka[1], Nayane dos Santos Brito Silva[8], Andreia da Silva Souza[8], Heloísa de Souza Andrade[8], Marília Rodrigues Silva Passos[8], Camila Ferreira Bannwart Castro[8,9], Celso T. Mendes-Junior[10], Rafael L. V. Mercuri[11,12,13], Thiago L. A. Miller[11,12], Jose Leonel Buzzo[11,12], Fernanda O. Rego[11], Nathalia M Araújo[14], Wagner CS Magalhães[14,15], Regina Célia Mingroni-Netto[1,2], Victor Borda[14], Heinner Guio[16,17], Carlos P. Rojas[16], Cesar Sanchez[16], Omar Caceres[16], Michael Dean[18], Mauricio L Barreto[19,20], Maria Fernanda Lima-Costa[21,22], Bernardo L Horta[23], Eduardo Tarazona-Santos[14,24,25,26], Diogo Meyer[2], Pedro A. F. Galante[11], Victor Guryev[7], Erick C. Castelli[8,9], Yeda A. O. Duarte[27,28], Maria Rita Passos-Bueno[1,2], Mayana Zatz[1,2†]

1. Human Genome and Stem Cell Research Center, University of São Paulo, São Paulo, SP, Brazil
2. Department of Genetics and Evolutionary Biology, Biosciences Institute, University of São Paulo, São Paulo, SP, Brazil
3. Hospital Israelita Albert Einstein, São Paulo, SP, Brazil
4. Instituto da Criança, Faculdade de Medicina da Universidade de São Paulo, São Paulo, SP, Brazil
5. Orthopedic Research Labs, Boston Children’s Hospital and Department of Genetics, Harvard Medical School, Boston, Massachusetts, United States of America.
6. Laboratório DASA, São Paulo, Brazil.
7. Laboratory of Genome Structure and Ageing, European Research Institute for the Biology of Ageing, University Medical Center Groningen, Groningen, Netherlands
8. São Paulo State University (UNESP), Molecular Genetics and Bioinformatics Laboratory, School of Medicine, Botucatu, State of São Paulo, Brazil.
9. São Paulo State University (UNESP), Department of Pathology, School of Medicine, Botucatu, State of São Paulo, Brazil.
10. Departamento de Química, Faculdade de Filosofia, Ciências e Letras de Ribeirão Preto, Universidade de São Paulo, Ribeirão Preto, São Paulo, Brazil.
11. Centro de Oncologia Molecular, Hospital Sirio-Libanes, São Paulo, Brazil.
12. Department of Biochemistry, Institute of Chemistry, University of São Paulo São Paulo, Brazil
13. Bioinformatics Graduate program, University of São Paulo, São Paulo, Brazil.
14. Departamento de Genética, Ecologia e Evolução, Instituto de Ciências Biológicas, Universidade Federal de Minas Gerais, Belo Horizonte, MG, Brazil.
15. Núcleo de Ensino e Pesquisa, Instituto Mário Penna, Belo Horizonte, MG, Brazil.
16. Laboratorio de Biotecnologia y Biologia Molecular, Instituto Nacional de Salud, Lima, Peru.
17. Universidad de Huánuco, Huánuco, Peru.
18. Division of Cancer Epidemiology and Genetics, National Cancer Institute, United States
19. Instituto de Saúde Coletiva, Universidade Federal da Bahia, 40110-040, Salvador, BA, Brazil.
20. Center for Data and Knowledge Integration for Health, Institute Gonçalo Muniz, Fundação Oswaldo Cruz, Salvador, BA, Brazil.
21. Instituto de Pesquisas René Rachou, Fundação Oswaldo Cruz, Belo Horizonte, MG, Brazil.
22. Programa De Pós-Graduação em Saúde Pública, Universidade Federal de Minas Gerais, Belo Horizonte, MG, Brazil.
23. Programa de Pós-Graduação em Epidemiologia, Universidade Federal de Pelotas, Pelotas, RS, Brazil.
24. Mosaico Translational Genomics Initiative, Universidade Federal de Minas Gerais, Belo Horizonte, MG, 31270-901, Brazil.
25. Facultad de Salud Pública y Administración. Universidad Peruana Cayetano Heredia, Lima, Peru.
26. Instituto de Estudos Avançados Transdisciplinares, Universidade Federal de Minas Gerais, Belo Horizonte, MG, 31270-901, Brazil.
27. Medical-Surgical Nursing Department, School of Nursing, University of São Paulo, São Paulo, SP, Brazil.
28. Epidemiology Department, Public Health School, University of São Paulo, São Paulo, SP, Brazil.

*Authors contributed equally

†Corresponding authors
mnaslavsky@usp.br
Rua do Matão, 277/211
ZIP 05508090
São Paulo - SP
Brazil
mayazatz@usp.br
Rua do Matão, Tv. 13, 106
ZIP 05508090
São Paulo - SP
Brazil

Supplementary Note 1: Cohort description 4
Supplementary Note 2: CEGH-Filter and variant analyses 6
Supplementary Note 3: Ancestry analyses 12
Supplementary Note 4: Clinical findings 14
Supplementary Note 5: Mobile Element Insertions (MEIs) 9
Supplementary Note 6: De novo assembly of non-reference sequences (NRS) 12
Supplementary Note 7: WGS Imputation 14
Supplementary Note 8: HLA 30
Supplementary Note 9: HLA Imputation 32
References 33

Supplementary Note 1: Cohort description

The Health, Well-being, and Aging (SABE) Study is a large effort to investigate health-related conditions of the elderly in Latin America and the Caribbean, initiated in 2000 with a follow-up design and at the time coordinated by the Pan American Health Organization. The Brazilian branch is based on the Public Health School at the University of São Paulo and enrolled elderly from the city of São Paulo, the largest in the Southern hemisphere. Subjects were invited based on probabilistic sampling from the census stratified from 60 years of age and older at the time of collection, with an oversample at the initial cohort of individuals with 75 and older. Every five years, recollection was performed with the inclusion of new cohorts (B, C, D) to reintroduce elderly subjects aging 60-64 (Supplementary Figure 1A)[3]. Supplementary Table 1 presents the age and sex distribution of SABE cohorts.

Supplementary Table 1. Age and sex distribution of SABE cohorts

Entry SABE cohort	Wave 2010 (n)	Unrelated individuals with WGS (n)	Age at collection (years ± s.d.)	Females (%)	Males (%)
A	746	640	78.89±6.82	416 (65)	224 (35)
B	240	220	65.52±1.29	135 (61.4)	85 (38.6)
C	349	311	61.86±1.36	193 (62.1)	118 (37.9)
Total	1335	1171	71.86±7.94	744 (63.5)	427 (36.5)

SABE participants were asked for specific consent on taking part in genomic studies from the year 2010 and beyond. All subjects in the genomic dataset have agreed on participating in this study on written consent forms approved by CEP/CONEP (Brazilian local and national ethical committee boards) under the following protocols: COEP FSP USP OF.COEP/23/10, CONEP 2044/2014, CEP HIAE 1263-10.

Our group in the HUG-CELL center was responsible for creating SABE DNA collection and sequencing its subjects to evaluate their genomes' features. In 2017, 609 individuals of three SABE cohorts (A, B, and C) were whole-exome sequenced, and variants and respective allelic frequencies deposited in ABraOM (http://abraom.ib.usp.br), a resource that has been widely used by the scientific community and by molecular diagnosis laboratories as controls (Supplementary Figure 1B)[4]. Later, whole-genome sequencing of near all samples from the 2010 wave was performed (Supplementary Figure 1, Supplementary Table 2).

From a total of 1,335 SABE participants enrolled in 2010, samples from 1,200 met quality criteria and were submitted to whole-genome sequencing at Human Longevity Inc. using the protocols previously described[5]. Relatedness was assessed by KING, and when identifying siblings and duos, one individual was maintained. The final number of unrelated individuals was 1,171 (Supplementary Figure 1, Supplementary Table 2).

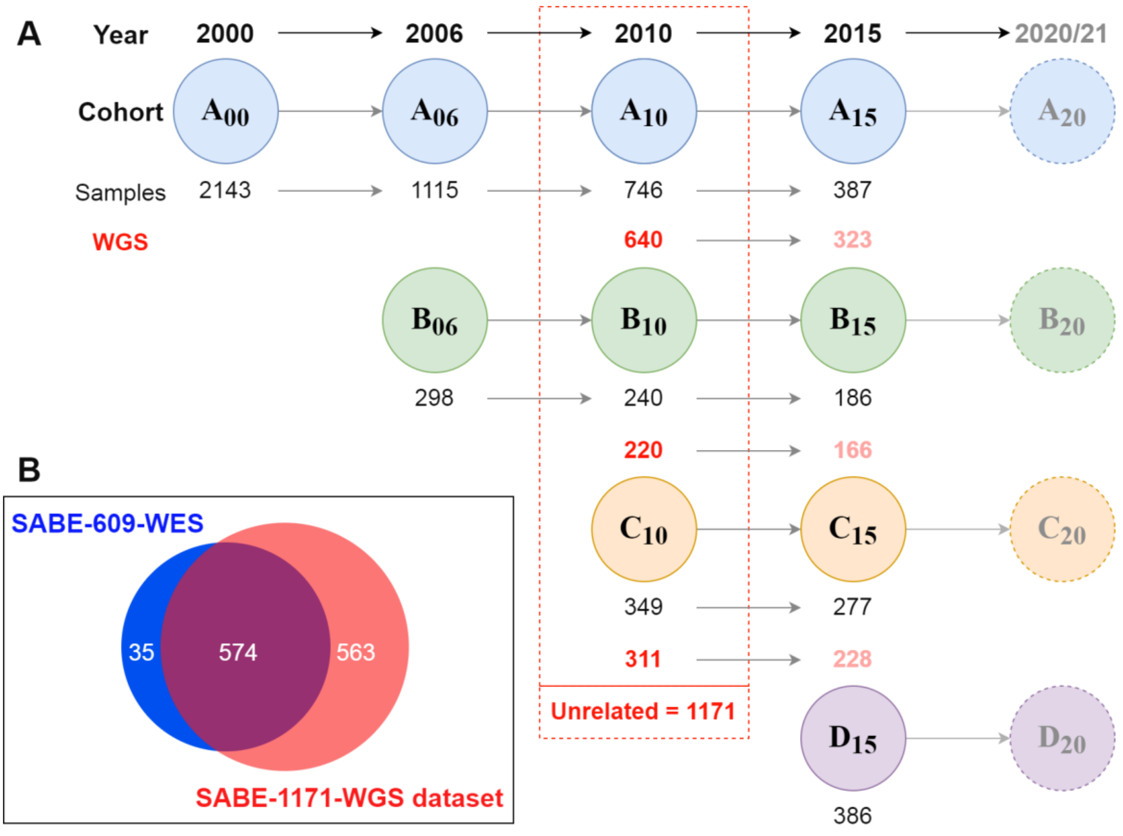

Supplementary Figure 1. **SABE cohorts longitudinal design and datasets deposited at ABraOM. A.** The first census-based cohort (A00) participants were enrolled in 2000, with 60 years of age and older, and followed up ever since in waves of phenotypic and biological samples recollections. A new cohort (B, C, D) was included every 5-6 years, with individuals aging 60-65 years old at enrollment. Whole-genome sequencing (WGS) was performed for most subjects (n=1200) of cohorts A, B, and C enrolled in the wave of 2010, of which 1171 are unrelated. **B.** Nearly half of SABE 2010 participants (N=609) were previously whole-exome sequenced, and this dataset of variants and allele frequencies was deposited at ABraOM. The current study refers to WGS of 1171 unrelated individuals, of which 574 were in the previously published dataset[4].

Since baseline, several health-related phenotypes were collected at their households, including the self-reported history of prevalent disorders, medications, measured anthropometric values, and functional tests relevant to elderly individuals. Questionnaires are comprehensive and were expanded and optimized every step, with about 3,500 variables, most nested within specific interrogations (treatment details on disorders). A total of 496 individuals were successfully recruited to perform additional data collections, including magnetic resonance (3T) of the brain at Albert Einstein Hospital (Supplementary Table 2).

Supplementary Table 2. List of SABE study collected phenotypes per cohort-year

Cohorts	Wave of collection	Measurements
SABE Cohort A	Baseline (2000-01)	Questionnaire;
		Anthropometry: weight, height, waist circumference and hips;
		Balance, mobility and flexibility. Cognitive test: "Mini" MMSE.
SABE Cohorts A+B	Follow up (2006)	Additional to above:
		MMSE;
		Blood pressure;
		Blood glucose.
SABE Cohorts A+B+C	Follow up (2010-12)	Additional to above:
		Wide range of haematological/biochemical blood tests;
		Serum frozen at -80ºC;
		HIV screening;
		Urinalysis (uri-color check);
		Immune response;
		Accelerometer (trace movement).
SABE Cohorts A+B+C	Genetics + MRI + Functional (2010-14)	DNA extraction of all collected in 2010-12;
		Whole-genome sequencing for 1,171 subjects of SABE cohorts A+B+C
		496 individuals were recruited to Albert Einstein Hospital to perform:<br>Brain MRI of n~452 (up to 5 acquisitions);<br>Pin pegboard of n~480;<br>Hand-grip strength n~480;<br>Ediburgh handedness inventory n~488;<br>Cognitive tests: 3MS and MMSE n~494;

Supplementary Note 2: CEGH-Filter and variant analyses

We performed a standard pipeline cited in the main Methods. All software and versions can be found in Supplementary Data 1.

In the final SABE dataset (1,171), WGS depth of coverage was assessed by GATK-DepthOfCoverage with a mapping quality threshold of 10 or greater[6]. Individual averages ranged from 31.3X to 64.8X, with a mean of individual averages of 38.65X and a median of 36.64X (Supplementary Fig. 2A). Horizontal coverage per vertical coverage thresholds yielded the complete dataset of 1,171 individual samples having 60% of bases with >30X and 91% of bases with >10X. A total of 1,098 individuals (93.7% of the sample) reach 70% of bases with >30X. (Supplementary Fig. 2B).

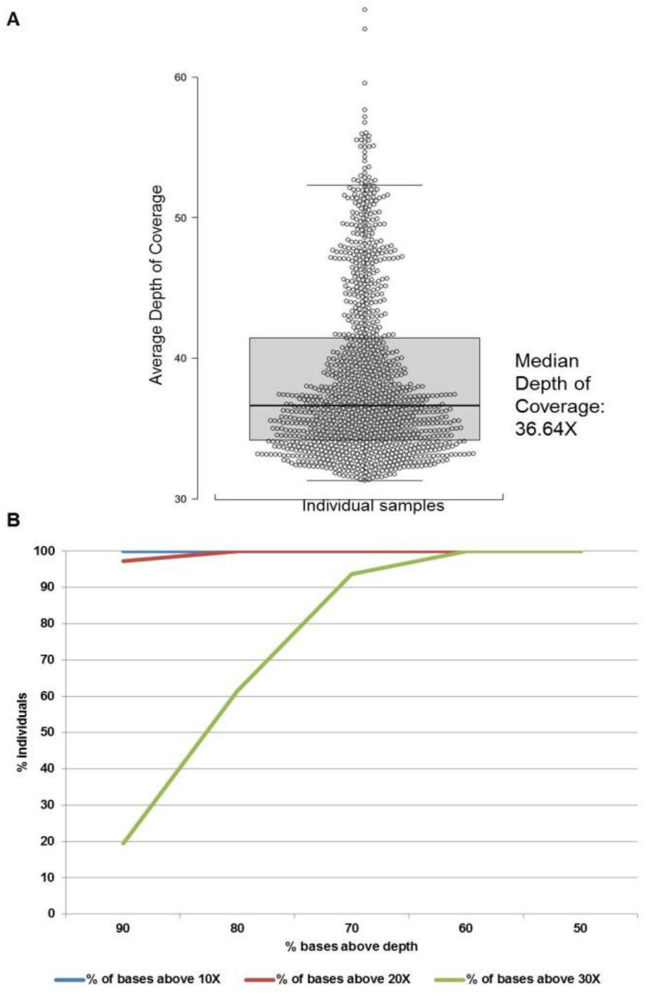

Supplementary Figure 2. Depth of coverage of 1,171 WGS samples from SABE. A. Distribution of the average depth of coverage per individual. The lower and upper hinges correspond to the 25th and 75th percentiles respectively, and the whiskers represent the 1.58 x inter-quartile range (IQR) extending from the hinges. **B.** Histograms of horizontal coverage per vertical coverage thresholds.

An in-house algorithm asserted genotype and variant qualities in addition to GATK flagging. CEGH-Filter (Supplementary Fig. 3) is a genotype walker algorithm that directly flags genotypes based on-site hard cutoff depth of coverage (DP>=10) and allele balance on a posteriori genotype calls (genotypes called heterozygous allelic proportion between inclusive 0.3 and 0.7; homozygous inclusive 0.1). After flagging all genotypes, each variant is flagged based on proportions of 'pass' genotypes carrying alternate alleles (heterozygotes 0/1 or alternative homozygotes 1/1) considering all genotypes at the site. Hard cutoffs on well-genotyped proportions of 90%, 50%, and one genotype to 10% will flag variants with 'Very Strong - vSR', 'Strong - SR' or 'Weak - WK' assertions, respectively. If no alternative allele carrying genotypes survive flagging, the variant is flagged either with 'Filtered out due to depth - FDP' or 'Filtered out due to allele balance - FAB' with corresponding proportions of each observation pending on 50% (FDP inclusive). If at least one genotype survives, but the quality of genotypes at the site does not fit quality criteria (cutting at 1,000 alleles or 500 genotypes), a 'Low cohort call' flag is added to vSR, SR, or WK. Allele frequencies are calculated before and after genotype flagging.

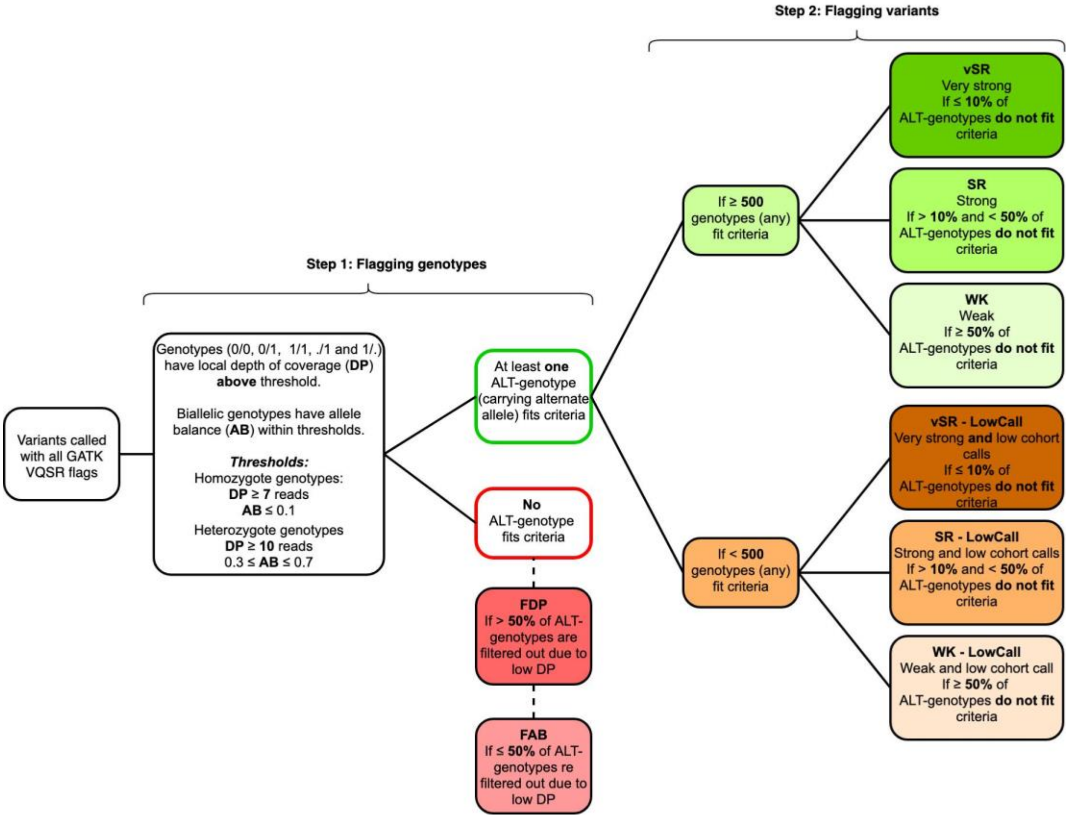

Supplementary Figure 3. CEGH-Filter algorithm. Steps, criteria, genotype flags, and variant flags.

Some genotypes are flagged by CEGH-Filter but not considered in the final counts to generate allele frequencies. Multiallelic informative genotypes (0/. and ./1), including SNVs, indels, and spanning deletions, are not included in allele frequency calculations due to dependency on other variants at the same site. Also, non-pseudoautosomal regions (non-PAR) loci along the X chromosome that harbor genotypes called as heterozygous state in male individuals are not accounted for, since they are expected to be hemizygous in those sites. These unexpected genotypes may be explained as false positives due to call errors or contamination, or else as true positives (duplications, aneuploidies, or mosaicism).

Although we consider further investigating those cases, at this point, we opted to exclude both multiallelic genotypes and non-PAR heterozygous in males from the counts and allelic frequency calculations, without, however, excluding the variant row per se. Therefore, some variants will appear to have zero allele frequency, even in non-FDP or non-FAB sites.

For overall counts (Supplementary Fig. 4), the high confidence dataset considered only GATK 'PASS' and CEGH 'vSR', 'SR', and 'WK'. Therefore, among the shadowed branch of counts, there are variants likely to be true positives, but rather fall within sites containing lower confidence calls. pLOF variants classified by LOFTEE[1] were considered irrespective of confidence label (HC or LC), since LC contains variants with at least one filter failed but can be a true positive. Additional evidence for nonsense-mediated decay or non-canonical splice sites can enrich the classification (https://github.com/konradjk/loftee). In the clinical analyses dataset, we have considered any CEGH-filter flag except for 'FDP' and 'FAB', since manual curation took place in the final step.

Annotation of variants per predicted function yielded the expected higher number of

intergenic (53.7%) and intronic (35.3%), whereas coding variants represent less than 1% with 635 thousand variants (Supplementary Table 3).

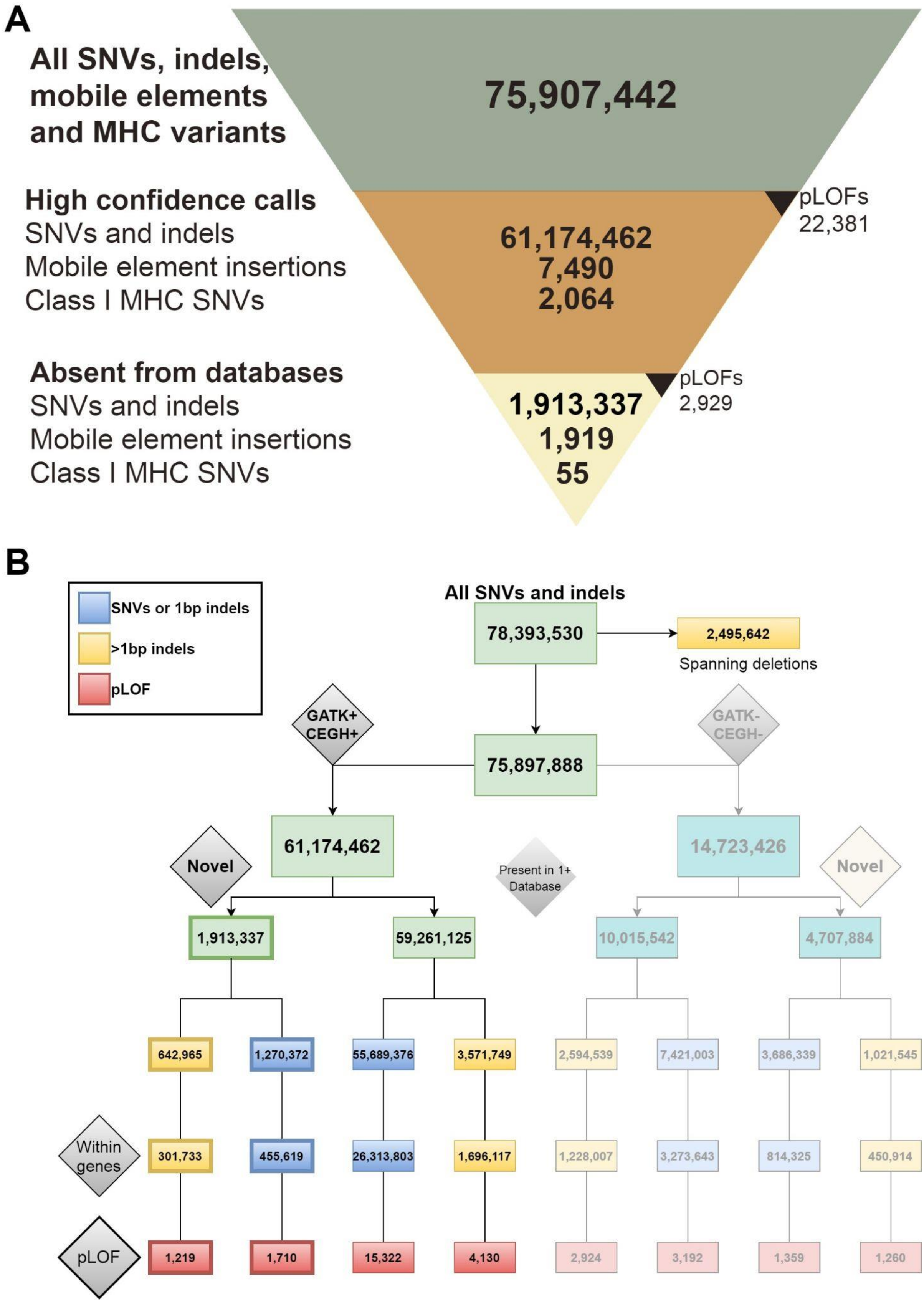

Supplementary Figure 4. **Single nucleotide variants (SNVs) and insertion/deletion (indel) counts in SABE WGS dataset.** Variant counts in SABE WGS dataset. **A.** Summary of all variants detected in SABE WGS dataset, including single nucleotide variants (SNVs), insertions/deletions (indels), mobile element insertions and

HLA remapped variants. High confidence flag is defined for SNVs and indels based on combined flags of GATK Filter and CEGH Filter. Variants with GATK Pass flags were counted as GATK+, and variants with CEGH vSR, SR, and WK flags were counted as CEGH+, a combination of both were considered high confidence (GATK+/CEGH+). SNVs and indels were absent from databases if not found in dbSNP v150, gnomAD v2.1.1 genomes, gnomAD v2.1.1 exomes, ESP6500 and 1000 Genomes. Mobile element insertions are absent from databases if not found in DGV and gnomAD. HLA variants are absent from databases if not found in IPD-IMGT/HLA Database version 3.4.0. **B.** Detailed total and high confidence counts for SNVs and indels in SABE WGS dataset. Spanning deletions were excluded from the annotation in the current study. High confidence calls are represented in the sharp color branch or else placed in the faded branch. Variants were classified as Novel (outer branches) if they are absent in all reference databases used for SNVs and indels (see in panel A), or if found in at least one database were included in inner branches. Yellow boxes represent counts of indels longer than 1bp, and blue boxes represent counts of SNVs and 1bp-indels. pLOF counts in red boxes are based on LOFTEE annotations[1].

Supplementary Table 3. Variant counts per predicted function.

Variants Predicted Function	Counts	% Group	% All
**Coding and splicing within genes**	**635689**		**0.81**
Nonsynonymous SNV	329744	51.87	0.42
Synonymous SNV	221436	34.83	0.28
Splicing	28526	4.49	0.04
Exonic;splicing	25752	4.05	0.03
Nonframeshift deletion	8218	1.29	0.01
Frameshift deletion	7196	1.13	0.01
Stopgain	6677	1.05	0.01
Nonframeshift insertion	4144	0.65	0.01
Frameshift insertion	3638	0.57	0.00
Stoploss	356	0.06	0.00
Splicing;splicing	2	0.00	0.00
**Noncoding within genes**	**29064505**		**37.08**
Intronic	27186280	93.54	34.68
UTR3	647982	2.23	0.83
Downstream	531982	1.83	0.68
Upstream	519497	1.79	0.66
UTR5	157192	0.54	0.20
Upstream;downstream	21185	0.07	0.03
UTR5;UTR3	379	0.00	0.00
Intronic;intronic	8	0.00	0.00
**Noncoding genes**	**4829729**		**6.16**
ncRNA_intronic	4544805	94.10	5.80
ncRNA_exonic	275495	5.70	0.35
ncRNA_exonic;splicing	4734	0.10	0.01
ncRNA_splicing	4669	0.10	0.01
ncRNA_UTR5	22	0.00	0.00
ncRNA_intronic;ncrna_intronic	4	0.00	0.00
**Intergenic**	**41363727**		**52.76**
Intergenic	41363715	100.00	52.76
Intergenic;intergenic	12	0.00	0.00
**Other**	**2499880**		**3.19**
Spanning deletion	2495642	99.83	3.18
NA + unknown	4238	0.17	0.01
**All**	**78393530**		

Supplementary Note 3: Ancestry analyses

In ancestry inference analyses, data from 30 populations were used as parental references, among which 16 were Native American (Supplementary Table 4).

Supplementary Table 4. Parental population used for ancestry inferences.

Population	Region/Parental Population	N	Reference
Luhya in Webuye, Kenya	Africa	99	Auton et al., 2015[7]
Yoruba in Ibadan, Nigeria	Africa	108	
Gambian in Western Divisions in the Gambia	Africa	113	
Mende in Sierra Leone	Africa	85	
Esan in Nigeria	Africa	99	
Han Chinese in Beijing, China	East Asia	103	
Southern Han Chinese	East Asia	105	
Chinese Dai in Xishuangbanna, China	East Asia	93	
Kinh in Ho Chi Minh City, Vietnam	East Asia	99	
Finnish in Finland	Europe	99	
British in England and Scotland	Europe	91	
Toscani in Italia	Europe	107	
Iberian Population in Spain	Europe	107	
Utah Residents (CEPH) with Northern and Western European Ancestry	Europe	99	
Aimara in Peru	Native American	11	Borda et al., 2020[2]
Ashaninka in Peru	Native American	33	
Awajun in Peru	Native American	22	
Candoshi in Peru	Native American	16	
Chopccas in Peru	Native American	7	
Lamas in Peru	Native American	17	
Matses in Peru	Native American	11	
Matsiguenka in Peru	Native American	3	
Mache in Peru	Native American	9	
Nahua in Peru	Native American	2	
Qeros in Peru	Native American	12	
Quechua in Peru	Native American	1	
Shimaa in Peru	Native American	23	
Shipibo in Peru	Native American	14	
Tallanes in Peru	Native American	30	
Uros in Peru	Native American	12	

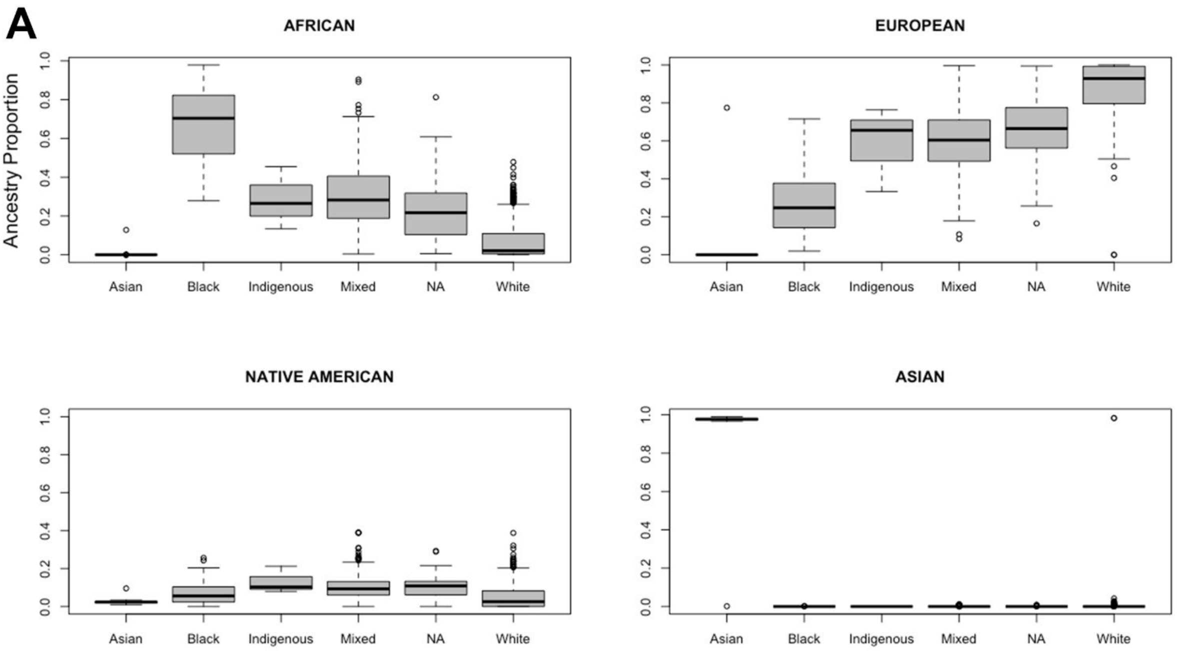

Self-reported ethno-racial group	N	Average ancestry AFR	EUR	NAM	EAS
Asian	32	0.004	0.024	0.025	0.947
Black	75	0.670	0.261	0.068	0.000
Indigenous	3	0.285	0.584	0.131	0.000
Mixed	330	0.298	0.600	0.102	0.000
NA = No answer/Not available	51	0.247	0.649	0.103	0.001
White	680	0.069	0.879	0.049	0.003

	Proportion	Number of individuals AFR	EUR	NAM	EAS
Individuals with ancestry proportion above or equal to	0.3	262	1072	8	33
	0.5	96	964	0	33
	0.7	46	711	0	33
	0.9	8	394	0	33

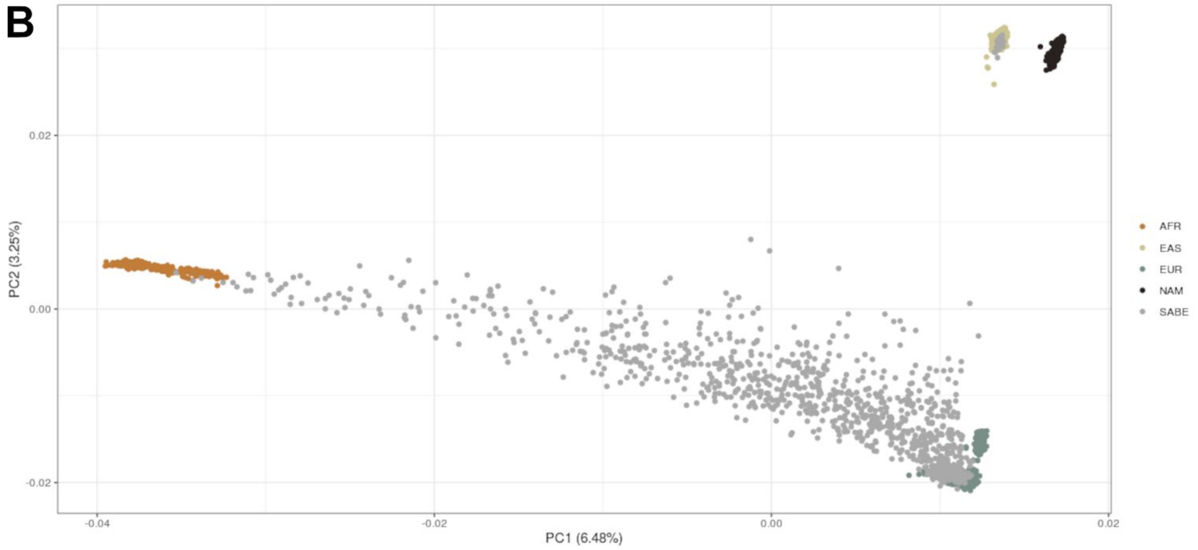

Supplementary Figure 5. Ancestry distributions and self-reported ethnoracial groups. A. Upper section: Boxplots of the proportions of genetic ancestry per self-reported ethno-racial groups (one way ANOVA p-value <2e-16; Tukey test p-value<0.001). The lower and upper hinges correspond to the 25th and 75th percentiles respectively, and the whiskers represent the 1.58 x inter-quartile range (IQR) extending from the hinges. Bottom table: Counts of individuals per self-reported ethno-racial

group and corresponding average ancestries; Number of individuals within different ranges of ancestry proportions. **B.** Principal component analysis of SABE individuals and parental populations. Analyses were performed with 372,527 SNVs (after overlapping- and LD-pruning). AFR, EAS and EUR from 1KGP3 and NAM from Borda et al., (2020)[2]. Three individuals self-reported as Indigenous had a high degree of admixture but were removed due to the small sample size of the group. Specific samples are described in Supplementary Table 4.

Supplementary Note 4: Clinical findings

4.1. Strategy

As initial classification criteria, we flagged all variants harbored by 4,250 OMIM disease genes (Supplementary Data 2) with ClinVar pathogenic assertions (Pathogenic, Likely Pathogenic, or Pathogenic/Likely Pathogenic) or predicted as promoting any loss of function consequence by LOFTEE algorithm. A total of 5,142 variants met the criteria (4,096 SNVs and 1,046 >1bp indels) (Pathogenicity analyses summarized in Supplementary Figure 6).

4.2. Frequencies of variants with potential clinical relevance

Although 10.6% of these variants are absent from population databases (gnomAD, dbSNP, and 1000 genomes), and most of which are indels, the remaining are mainly rare single-nucleotide substitutions (frequencies ≤ 0.001) (Supplementary Table 5).

Supplementary Table 5. Distribution of variants identified in SABE 1171 cohorts with potential clinical relevance in OMIM Disease genes absent and present in database, per frequency

			**Count per frequency bins of variants absent from population databases (% Singleton)**				
**Type (total counts)**	**Category**	**Counts**	**≤ 0.001**	**≤ 0.01**	**≤ 0.05**	**≤ 0.1**	**> 0.1**
SNV Substitution (25)	Any assertion on ClinVar	23	23 (96)	0	0	0	0
	Any pathogenic assertion on ClinVar*	2	2 (100)	0	0	0	0
	pLOF	25	2 (100)	0	0	0	0
	pLOF & Pathogenic assertion on ClinVar*	2	2 (100)	0	0	0	0
1bp-INDELs (293)	Any assertion on ClinVar	9	9 (100)	0	0	0	0
	Any pathogenic assertion on ClinVar*	8	8 (100)	0	0	0	0
	pLOF	292	289 (97)	3 (33)	0	0	0
	pLOF & Pathogenic assertion on ClinVar*	7	7 (100)	0	0	0	0
INDELs >1bp (229)	Any assertion on ClinVar	9	9 (100)	0	0	0	0
	Any pathogenic assertion on ClinVar*	9	9 (100)	0	0	0	0
	pLOF	229	214 (54)	9 (0)	0	1 (0)	3 (0)
	pLOF & Pathogenic assertion on ClinVar*	9	9 (100)	0	0	0	0
			**Count per frequency bins of variants present in at least one population database (% Singleton)**				
**Type (total counts)**	**Category**	**Counts**	**≤ 0.001**	**≤ 0.01**	**≤ 0.05**	**≤ 0.1**	**> 0.1**
SNV Substitution (3151)	Any assertion on ClinVar	1646	1156 (77)	386 (0)	52 (0)	13 (0)	37 (0)
	Any pathogenic assertion on ClinVar**	953	796 (83)	142 (0)	6 (0)	2 (0)	**5 (0)**
	pLOF	2047	1614 (86)	289 (0)	59 (0)	13 (0)	71 (0)
	pLOF & Pathogenic assertion on ClinVar**	289	257 (85)	32 (0)	0	0	0
1bp-INDELs (627)	Any assertion on ClinVar	195	115 (74)	54 (0)	10 (0)	2 (0)	14 (0)
	Any pathogenic assertion on ClinVar**	87	72 (75)	15 (0)	0	0	0
	pLOF	619	373 (77)	144 (0)	53 (0)	10 (0)	40 (0)
	pLOF & Pathogenic assertion on ClinVar**	81	68 (75)	13 (0)	0	0	0
INDELs >1bp (817)	Any assertion on ClinVar	217	131 (71)	60 (0)	8 (0)	3 (0)	13 (0)
	Any pathogenic assertion on ClinVar**	92	83 (77)	8 (0)	1 (0)	0	0
	pLOF	788	421 (72)	242 (1)	62 (0)	15 (0)	41 (0)
	pLOF & Pathogenic assertion on ClinVar**	74	69 (78)	5 (0)	0	0	0

*Pathogenic, Likely pathogenic or Conflicting interpretations of pathogenicity with at least one P or LP assertion

**Pathogenic or Likely pathogenic. Excludes any variants asserted as Conflicting interpretations of pathogenicity

Few exceptions reach over 0.1 Among these high-frequency variants, we highlight the five asserted as pathogenic (Supplementary Data 3): (a) rs429358 *APOE* p.C130R (NM_000041) with a frequency of 0.13, which should be considered in phase with rs7412 p.R176C to the well-known functional haplotypes (ε2, ε3, and ε4) associated with late-onset Alzheimer's and type III hyperlipoproteinemia; **C.** rs17261572 and rs1566734, which were asserted as pathogenic before large allelic frequency datasets and community-based consensual criteria such as recommendations provided by American College of Human Genetics and Genomics (ACMG) were available[8]; and (c) variants classified as risk factors (lower penetrance by definition) in sporadic breast cancer multifactorial susceptibility (rs2046210) or in glycine metabolism on a digenic model of inheritance (rs35329108). Therefore, pathogenic assertions should be considered with caution.

4.3. Context of variants

These 5,142 variants fall within 1,949 unique genes and 168 intergenic regions or overlapping genes, 98% of which harbor no more than 10 variants (Supplementary Table 6). Most genes are annotated as either recessive or non-monogenic modes of inheritance, but a considerable amount of genes (749) are described as having either dominant or both dominant and recessive inheritance (Supplementary Table 7).

Supplementary Table 6. Number of genes and regions that harbor one or more variant with potential clinical relevance

Variants per gene/region	Number of genes/regions	%
1	992	46.86
2	493	23.29
3	257	12.14
4	135	6.38
5	81	3.83
(5-10]	126	5.95
(10-20]	26	1.23
>20	7	0.33
**Total**	2117	100

Supplementary Table 7. Number of genes that harbor variants with potential clinical relevance per inheritance mode

Inheritance mode (OMIM)	Number of genes
AD/XLD only	437
AR/XLR only	1170
AD and AR, XLD and XLR	312
Somatic mutation	31
Multifactorial	12
Mitochondrial	6
Digenic (Dominant or Recessive)	20
Somatic mosaicism	1
**Total**	1989

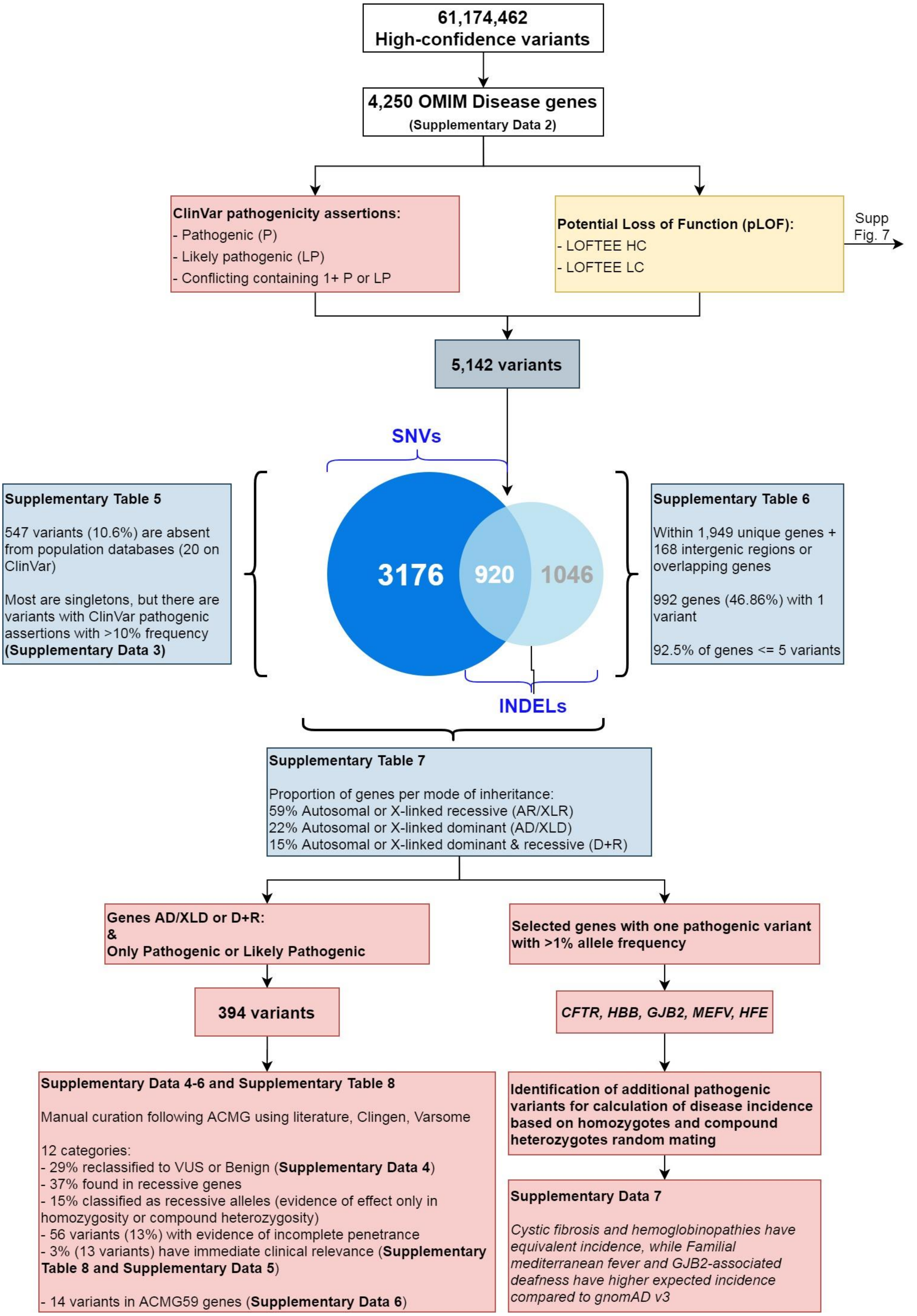

Supplementary Figure 6. Filtering strategies for the identification of variants of potential clinical relevance and indication of downstream results. Among high-confidence variants, we have identified a total of 5,142 variants within 4,250 OMIM disease genes (Supplementary Data 2) that were found to have pathogenic, likely pathogenic, or conflicting containing at least one pathogenic ClinVar assertions, or classified as potential loss of function (pLOF).

Downstream analyses pointed that: over 10% are absent from population databases, but most are singleton or low frequency (Supplementary Table 5; Supplementary Data 3); most genes contain up to five variants (Supplementary Table 6); most genes are annotated to associate with recessive modes of inheritance (Supplementary Table 7); manual curation of variants initially classified as pathogenic or likely pathogenic in genes of dominant inheritance can be either reclassified or fall indeed in recessively inherited conditions or else are required to be in trans with a more deleterious variant (Supplementary Data 4-5, Supplementary Table 8); 14 pathogenic variants harbored by ACMG-59 genes were identified (Supplementary Data 6); SABE cohort recapitulates variant-based incidence of recessive disorders that are more prevalent in European or African populations (Supplementary Data 7).

4.4. Manual curation of variant pathogenicity on genes associated with dominant mode of inheritance

In order to identify individuals carrying variants with potential clinical implications, including the reassessment of related phenotypes to support the analyses, we have filtered a total of 394 variants asserted as either 'Pathogenic' or 'Likely Pathogenic' (P/LP) in genes annotated to have a dominant mode of inheritance only, and in genes with more than one mode of inheritance, including dominant or monoallelic. Manual curation aiming reclassification of pathogenicity using ACMG criteria was performed by two independent clinical geneticists (professionals with clinical genetics residency and previous experience in clinical exome analysis and variant pathogenicity classification using ACMG criteria). Manual curation included functional studies and segregation information described in the available literature, evidence details on the original assertions, and allele frequency. Each of the 394 variants in dominant genes containing P/LP ClinVar assertions was submitted to manual curation aided by population-specific frequencies (gnomAD and SABE, mainly); ClinGen (to reannotate inheritance modes); review of VarSome automated calculation of ACMG classification criteria; and in-depth analyses on ClinVar submissions leading to classification, particularly in evidence levels (ACMG criteria assigned and provided by submitters, to adjust PP5), details on co-segregation of ClinVar assertion combined with literature reports of carriers and families (to adjust PP1). OMIM aided reclassification of gene's mode of inheritance in cases where ClinGen information could not be conclusive, such as only one affected case was reported and recessive mode could not be excluded. When loss of function consequence would only be detected in trans with another P/LP variant and not by itself (hypomorphic variants) the allele did not meet criteria for haploinsuficiency and dominant phenotype (hereby classified as 'recessive allele'). A total of 116 variants (29%) were reclassified as non-pathogenic assertions (benign, likely benign or unknown significance) (Supplementary Data 4), most of which had no assertion criteria provided (70 variants), 41 had criteria provided by a single submitter and 5 by multiple submitters. The remaining 278 kept as pathogenic or likely pathogenic (Supplementary Table 8). Among the latter, literature validation and matching phenotypes, when available, enabled further characterization of variants to either a reported reduced penetrance, non-dominant mode (of the specific allele or gene), or associated to clinical features that are not severe enough to cause mortality before the average age of subjects (Supplementary Table 8, Supplementary Data 5).

Supplementary Table 8. Counts of variants per category after manual curation of 394 pathogenic variants in genes with dominant mode of inheritance

**Category after curation**	**Counts**	**%**
Compatible finding	2	0.51
Subclinical/Mild phenotypes	6	1.52
Somatic mosaicism	1	0.25
Somatic mosaicism/Subclinical/Mild phenotype	2	0.51
Conditional (PGx)	2	0.51
Incomplete penetrance/Subclinical/Mild phenotypes	13	3.30
Recessive allele/Incomplete penetrance/Subclinical	4	1.02
Incomplete penetrance	39	9.90
Recessive allele/Incomplete penetrance	2	0.51
Recessive allele	61	15.48
Recessive gene	146	37.06
Reclassified	116	29.44
**Total**	394	100

4.5. Manual curation of variant pathogenicity on ACMG 59 actionable genes

We also analyzed P/LP variants in 59 actionable genes following ACMG recommendation[9] and found 14 variants distributed in heterozygosity in different individuals all in the heterozygous state (Supplementary Data 6), among which *BRCA2* and *RYR1* harbor four variants each. Ten variants were classified using the above-mentioned protocol as pathogenic with reported incomplete penetrance; three were described as pathogenic only when in *trans* with another pathogenic variant (recessive alleles), and one potential phenotypic match (outcome compatible with finding) in *LDLR*.

4.6. Assessment of variant-based incidence pathogenicity on selected genes associated with recessive mode of inheritance

To roughly estimate the incidence using counts of heterozygotes from SABE and gnomAD global and population-specific datasets, we selected five genes associated with prevalent monogenic clinical phenotypes: cystic fibrosis (*CFTR*), hemoglobinopathies (*HBB*), deafness (*GJB2*), familial Mediterranean fever (*MEFV*), and hemochromatosis (*HFE*) (Supplementary Data 7). These genes were used to filter high frequency (up to 5%) and low frequency (including singletons) known pathogenic variants, as classified by respective Locus Specific Databases. For CFTR we have used CFTR2 (https://cftr2.org/); for HBB, HbVar (http://globin.cse.psu.edu/hbvar/menu.html); for GJB2, Deafness Variation Database (http://deafnessvariationdatabase.org/); for MEFV, Infevers (https://infevers.umai-montpellier.fr/web/); and for HFE, LOVD-HFE (https://databases.lovd.nl/shared/genes/HFE). The same variants were searched in gnomAD v3 and counts per population were used to calculate a frequency per population (number of genotypes fixed at 71700). Incidence was calculated without correction for penetrance, assuming panmixia and even distribution between sexes. We combined counts of heterozygotes (independently for each variant within a locus, as observed) and number of individuals. The fraction of carriers within each sample

was squared (providing a fraction of possible couples of carriers) and divided by four (an offspring of 25% of compound heterozygotes or homozygotes).

4.7. Distribution of potential loss of function variants within OMIM Disease genes

There are 4,000 potential loss of function (pLOF) variants in SABE that fall within OMIM Disease genes, of which 3,704 are 'non-Benign', which excludes ClinVar benign, likely benign, or conflicting assertions that lack pathogenic entries (Supplementary Fig. 7). We have found a normal distribution of individual loads of pLOF variants in heterozygous state (Supplementary Fig. 8A) and homozygous state (Supplementary Fig. 8B), and a Poisson distribution of variants with one or more pathogenic assertions (Supplementary Fig. 8C), with medians of 55, 25, and 1 variants per person, respectively. A comparison of allele frequencies of these variants between SABE and gnomAD revealed a high correlation regardless of pLI contexts (Supplementary Fig. 9A).

Assuming a higher proportion of false positives among indels, mainly longer ones, we have filtered only pLOF variants produced by single nucleotide variants (substitutions or 1bp long indels), with allele frequency of 5% or lower on SABE and flagged as high-confidence by LOFTEE. These strict filtering criteria yielded a total of 1,853 pLOF variants, which were Poisson distributed with a median of 5 in heterozygosity per individual (Supplementary Fig. 8D), a median of 0 in homozygosity (although 20% have one or two, Supplementary Fig. 8E) and a median of 0 with one or more pathogenic assertion (39% have one to four, Supplementary Fig. 8F). A high correlation with gnomAD frequencies is maintained in this subset (Supplementary Fig. 9B). Further detailed analysis of pLOF variants within genes of pLI ≥ 0.7 with higher differences in allele frequencies showed that they were either long indels multiallelic or homopolymers flagged as low confidence or in low-quality sites in gnomAD. Also, annotation of variants in intergenic regions may be wrongly attributed and lead to spurious flagging (Supplementary Fig. 9C). Therefore, regardless of the dataset quality cutoff, we have found non-deviant frequencies as compared with gnomAD.

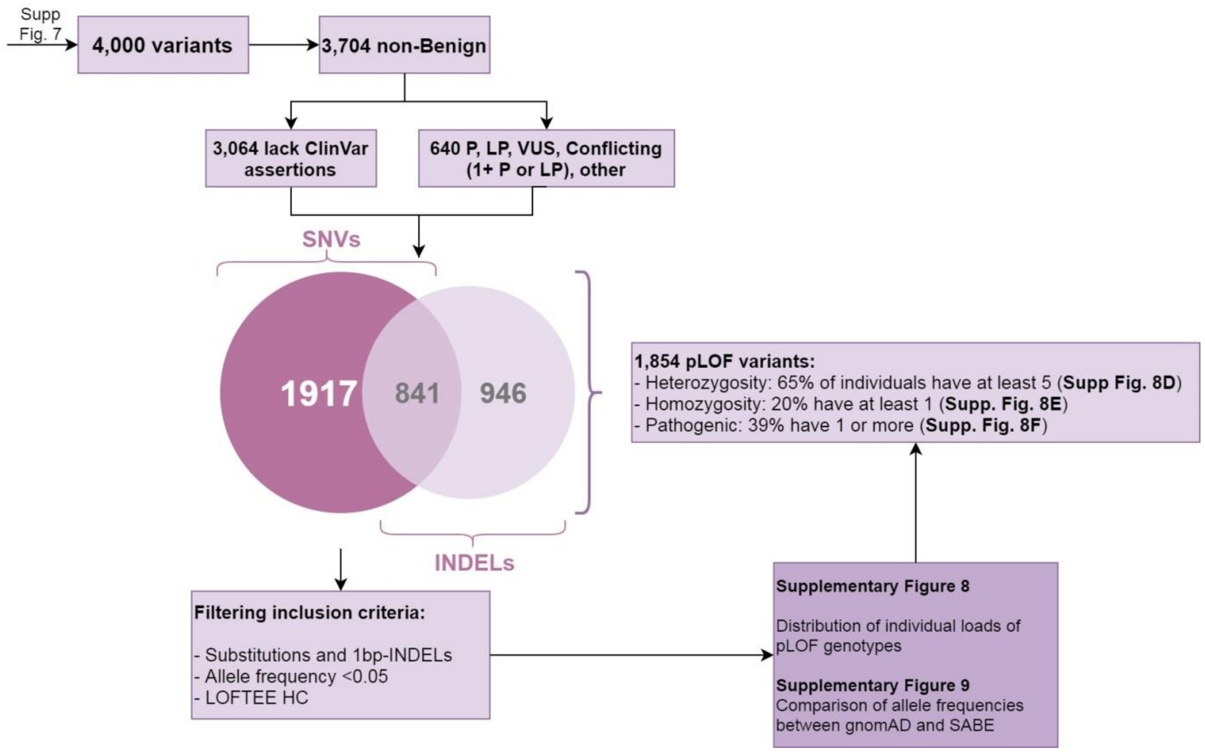

Supplementary Figure 7. Filtering strategies for identification of variants of potential loss of function. Among high-confidence variants, we have identified 5,142 variants within 4,250 OMIM Disease genes, 4,000 of which were classified as potential loss of function (pLOF). A subset of any pLOF non-Benign variants corresponds to 3,704 variants with any ClinVar non Benign assertion (640 variants) plus 3,064 variants that lack any assertions. Substitutions, 1bp-indels, and indels >1bp were analyzed for the distribution of individual loads (Supplementary Fig. 8A, 8B, and 8C) and allele frequencies compared to gnomAD (Supplementary Fig. 9A). Further filtering to remove indels >1bp, common variants and LC-flagged pLOFs yielded 1,854 variants, which individual load distributions were also analyzed (Supplementary Fig. 8D, 8E, and 8F) as well as frequency comparison to gnomAD (Supplementary Fig. 9B).

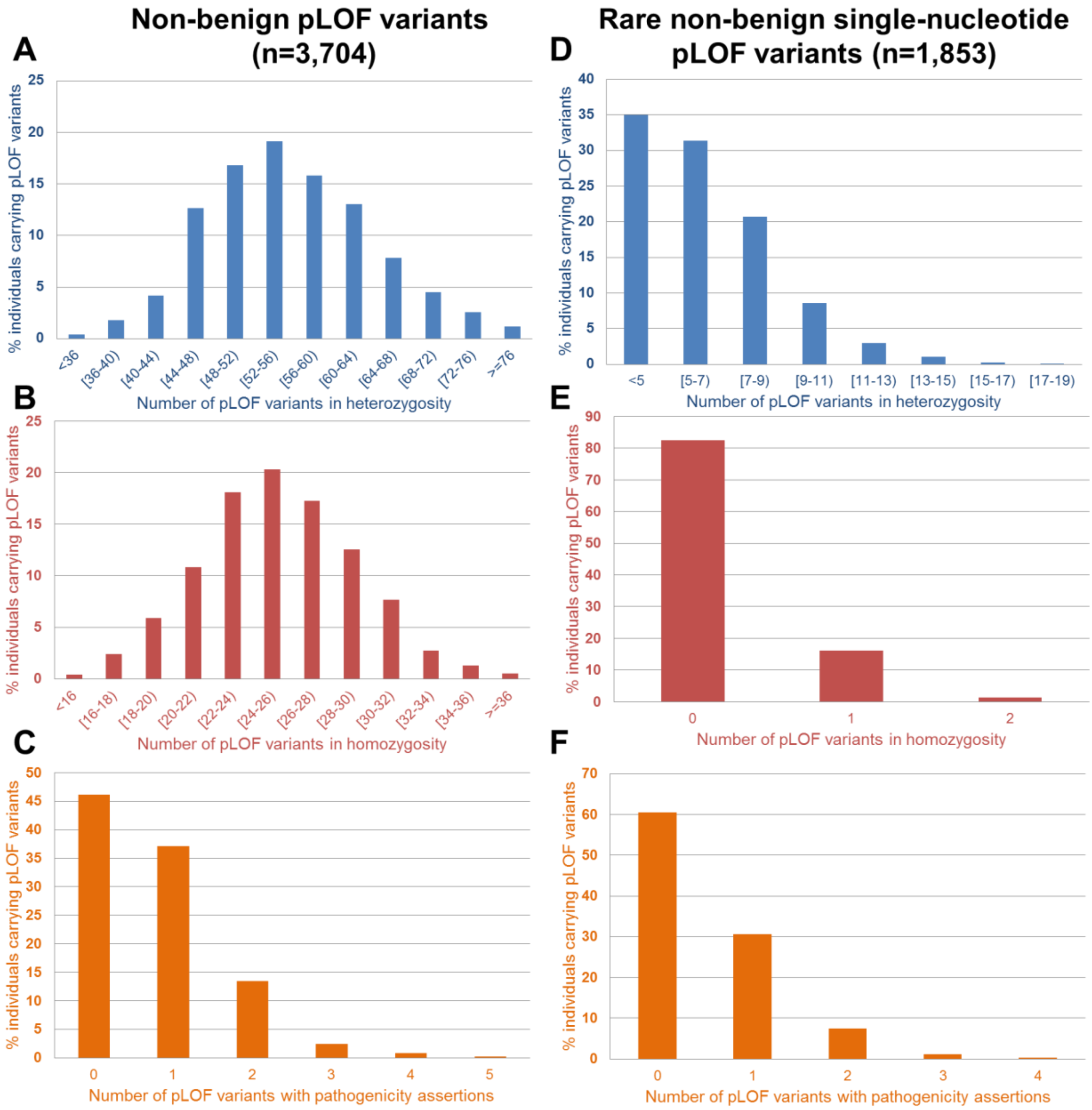

Supplementary Figure 8. Distribution of individual loads of potential loss of function (pLOF) variants. Left panels: a subset of any non-Benign pLOF (ClinVar non-Benign assertions plus variants that lack any assertions) variants. Histogram of individual loads of pLOF variants in (**A)** heterozygosity, (**B)** homozygosity, and (**C)** variants with pathogenic assertions on ClinVar (including Pathogenic, Likely Pathogenic and Conflicting containing one or more pathogenic entries). Right panels: a subset of pLOF variants that are single nucleotide substitutions or 1bp-indels below 5% SABE cohort frequency and flagged as LOFTEE HC. Histogram of individual loads of pLOF variants in **(D)** heterozygosity, **(E)** homozygosity, and **(F)** variants with pathogenic assertions on ClinVar.

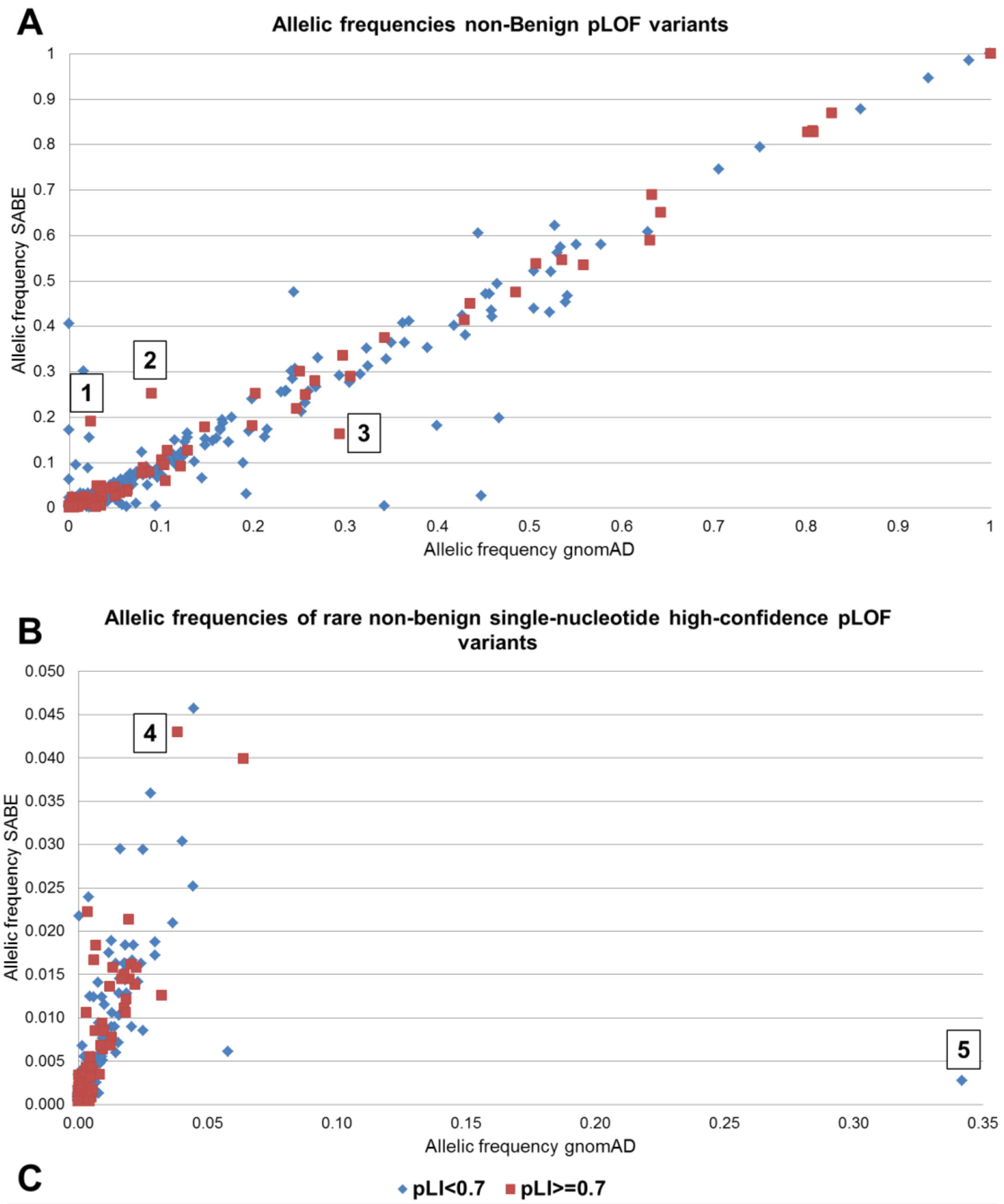

C

# in Plot	Filter	Chr	Position	Ref	Alt	Gene	pLI	Allele frequencies SABE	Allele frequencies gnomAD v2.2	Cause of disparities
1	All pLOF Non Benign	9	128694106	-	TATA	*SET*	0.96	0.1905	0.0237	Multiallelic INDEL with Low Confidence
2	All pLOF Non Benign	12	70353914	A	-	*CNOT2*	1.00	0.2516	0.0893	Multiallelic INDEL in poly-A Homopolymer
3	All pLOF Non Benign	16	89233469	TGCAACTCAACT CACACTGCGTA	-	*ANKRD11 ;ZNF778*	1.00;0	0.1619	0.2939	Intergenic, pLI from neighbor gene
4	Rare HC pLOF SNVs	X	153481313	G	-	*BGN; HAUS7*	0.92; 0.96	0.0430	0.0385	pLOF annotation incorrect
5	Rare HC pLOF SNVs	3	10046724	G	-	*FANCD2*	0.00	0.0027	0.3422	Low quality site (absence of homozygotes)

Supplementary Figure 9. Comparison of allele frequencies between pLOFs found in SABE and gnomAD (v2.2), within genes of pLI>=0.7 and pLI<0.7. **A.** Subset of non-Benign variants provided a comparison of pLOF frequencies of up to 100%. **B.** Rare single nucleotide variants flagged as HC on LOFTEE. **C.** Five examples of deviation were manually verified in gnomAD and explained by context leading to calls or annotations.

Supplementary Note 5: Mobile Element Insertions (MEIs)

As presented in the main text and methods, mobile element insertions (MEIs) were identified across all samples and annotated by element types and frequency groups. Regarding their genomic locations, the correlation between number of MEIs and chromosome length can be observed in Supplementary Figure 10. The genomic context regarding AT/GC content in relation to MEI events per category can be observed in Supplementary Figure 11.

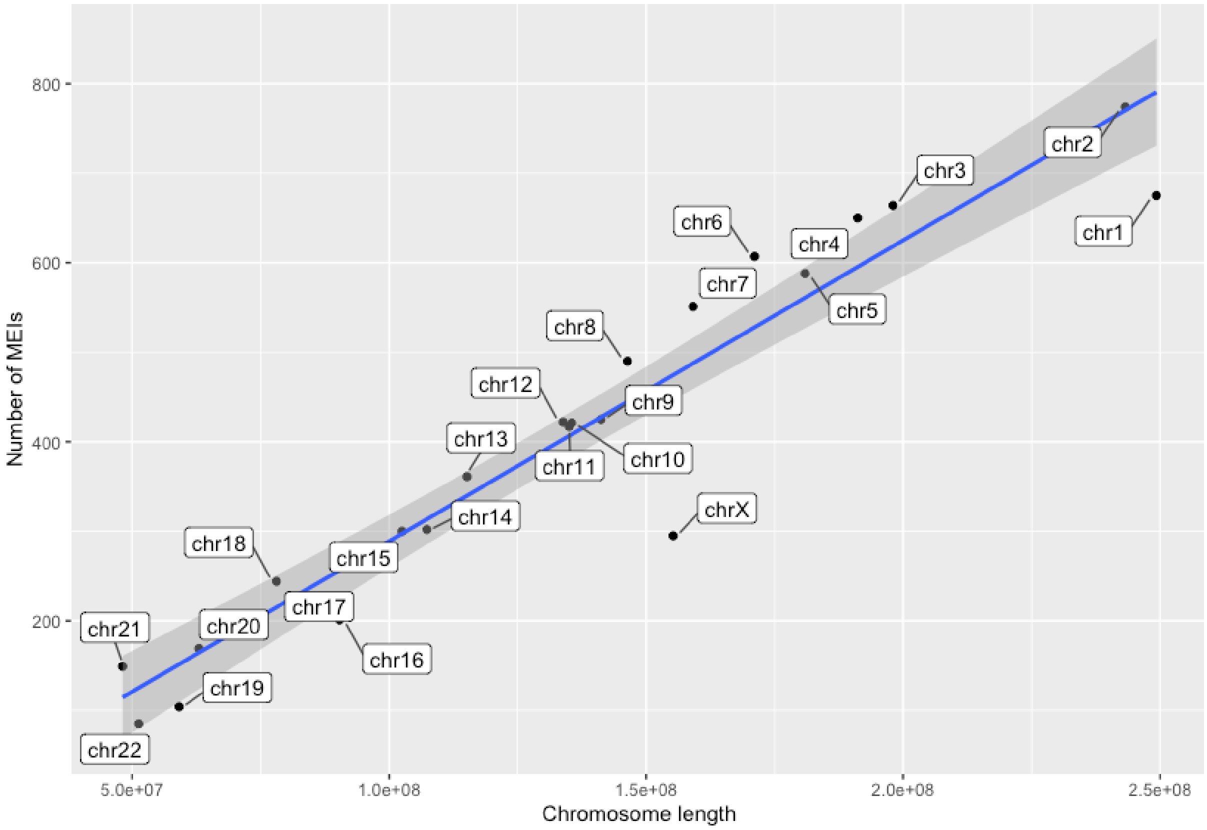

Supplementary Figure 10. Number of MEIs per chromosome length. We observed a positive correlation between the number of MEIs and the chromosome length (one-sided test, not corrected for multiple tests, p-value = 2.74e-6; rho = 0.95; Spearman's rank correlation; d.f. = 21). Shaded area represents the standard error.

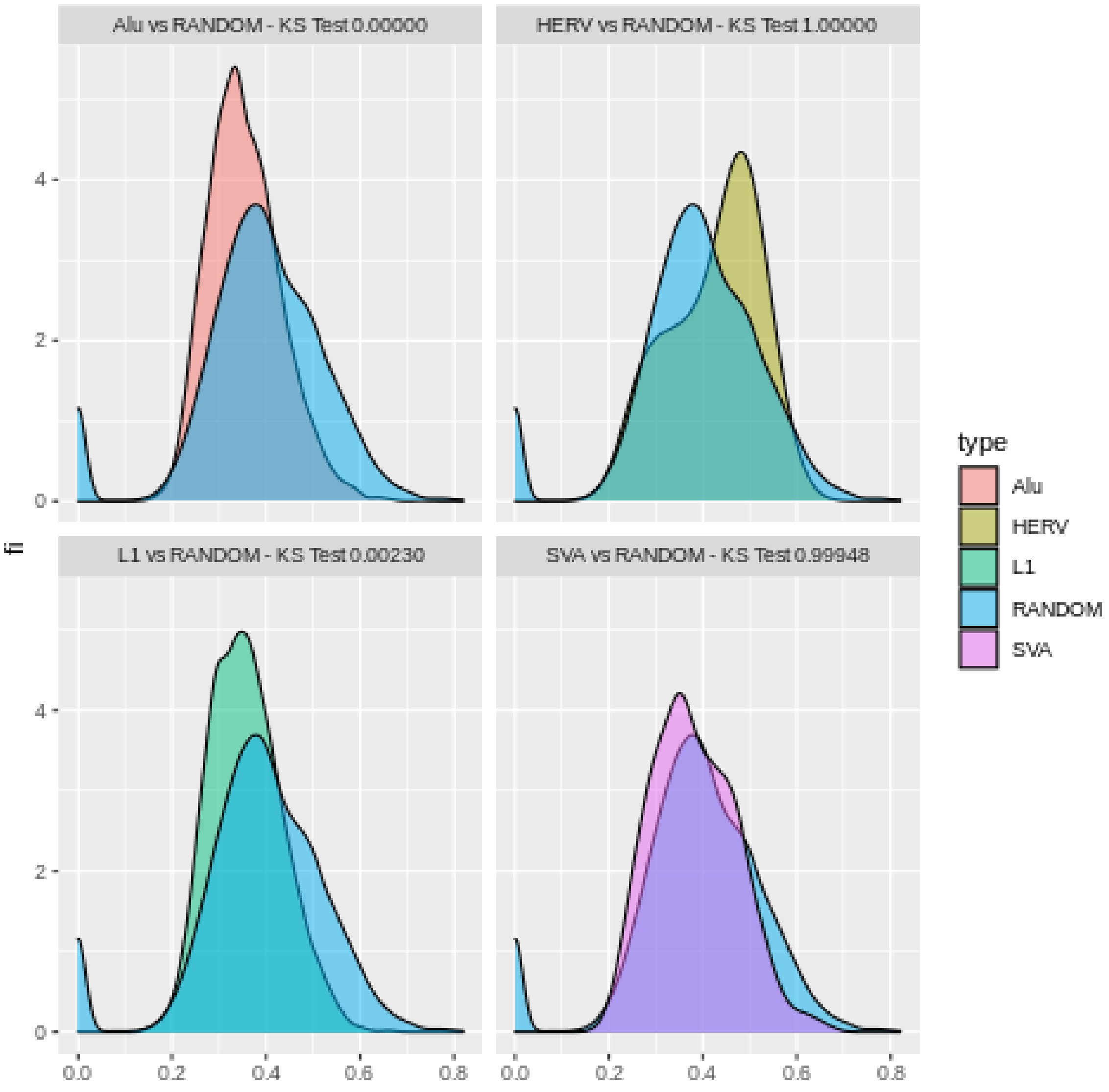

Supplementary Figure 11. Base pair composition of mobile elements insertion point. We randomly selected 10,000 windows of length 100 bp from the human genome version 38 and calculated their GC content. Then, we made the same for all Mobile Elements Insertion points, discriminating by *Alu*, L1, SVA, and HERV. Finally, we tested with Kolmogorov-Smirnov test (KS test) the random windows distribution against those of MEIs. L1 and *Alu* insertions are skewed to AT-rich regions, while HERVs are biased to GC-rich regions.

Next, we have annotated the overlaps between occurrence of MEI events in OMIM disease genes (Supplementary Data 2) and their respective genomic contexts. Results can be found in Supplementary Table 9 (total counts) and Supplementary Data 8 (MEI events in exonic regions of OMIM Disease genes).

Supplementary Table 9. Counts of mobile element insertions per frequency group, genomic context and OMIM annotation

		**Genomic context of MEIs**					
		**Exon**		**Intron**		**Flank**	
**Frequency group**	**Count of MEI events***	**All**	**OMIM gene**	**All**	**OMIM gene**	**All**	**OMIM gene**
**Shared**	2111	69	15	1949	561	93	0
**SABE Private or Singleton**	725	24	4	664	191	37	1
**Total**	2836	93	19	2613	752	130	1

*Events are counted when MEIs occurring in genes are unique, therefore these numbers do not account for frequency (when more than one individual carry MEIs spanning the same genomic position)

Regarding MEIs identified in exonic regions of OMIM genes (Supplementary Data 8), all genes but *HCN1*, *PACS1* and *PIK3R1* are associated with recessive disorders, susceptibility loci or non-disease traits. *HCN1* variants associated with AD epilepsy are all missense with gain of channel function or dominant negative effects even though pLI in gnomAD is 1, multiple controls in Developmental Delay Database (DDD) have been identified with intragenic exon spanning deletions. Schuurs-Hoejimakers is associated with a single recurrent variant in *PACS1* (NM_018026.4: c.607C>T), and even though pLI in gnomAD is also 1, loss of function variants (or deletions) in this gene have never been reported associated with disease in humans. *PIK3R1* variants have been associated both with AR inheritance (loss of function, nonsense, variant) and AD (splicing, predicted gain of function, variants), pLI in gnomAD is 0.02. Therefore there is evidence that none of the MEI in OMIM genes that could potentially lead to truncation of the gene product (and a loss of function consequence) are likely to be associated with a severe disease phenotype in the individuals from SABE cohort.

Supplementary Note 6: De novo assembly of non-reference sequences (NRS)

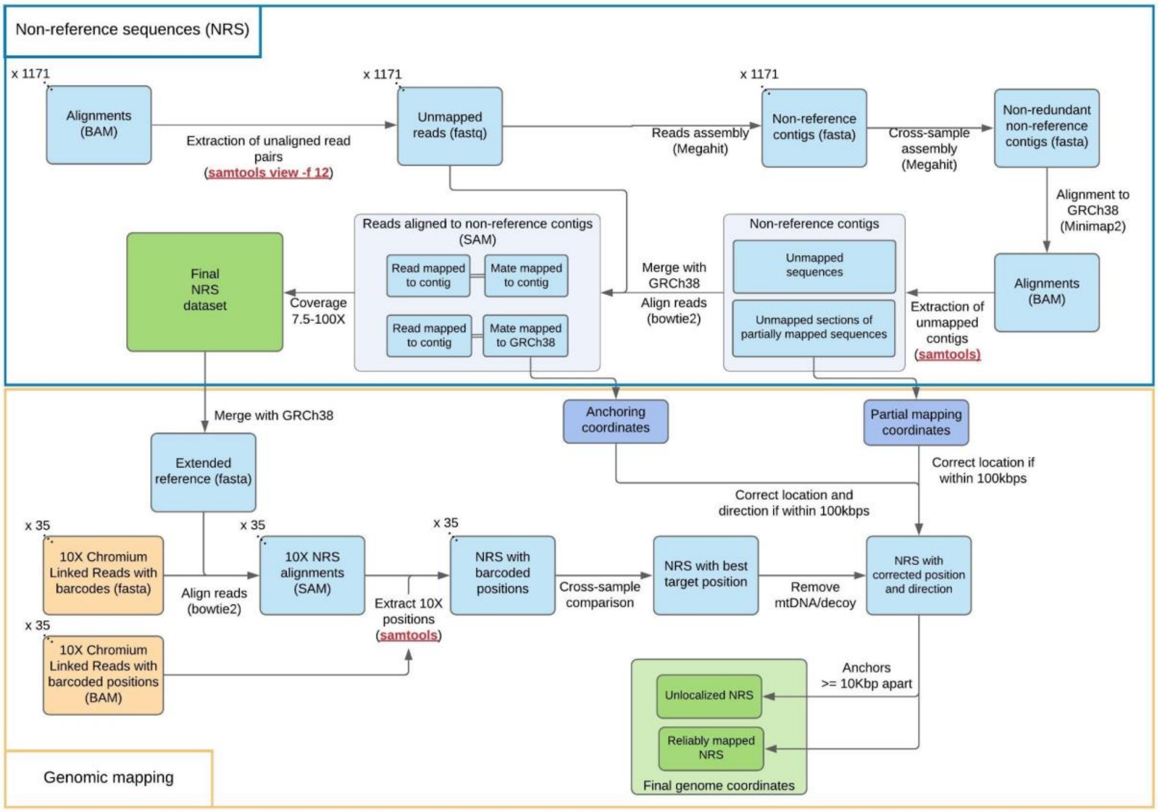

Supplementary Figure 12. Flowchart representing the non-reference sequences pipeline as described in Methods section.

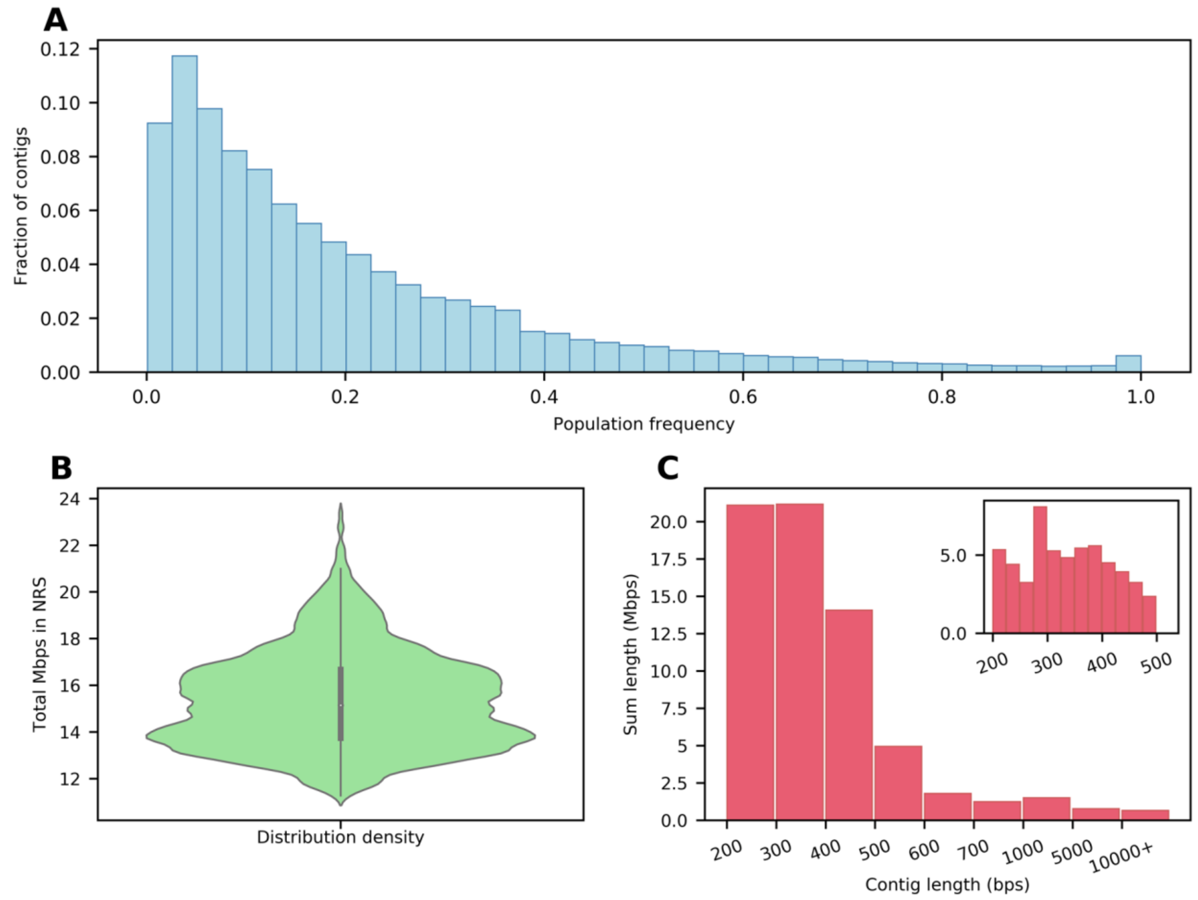

Supplementary Figure 13. Analysis of non-reference contigs. A. The frequency of non-reference contigs (NR-contigs) in the SABE population. There are 372 NR- contigs found in all samples in the population. **B.** Violin plot showing the distribution of the total NR-contigs length in megabase pairs (Mbps) for the individuals. **C.** Length distribution of the NR-contigs with the vertical axis representing the sum of the contig lengths. From a total of 67Mbps of NR-contigs, 56Mbps are less than 500 base pairs long. There are 40 NR-contigs longer than 10kbps.

Supplementary Note 7: WGS Imputation

Supplementary Table 10. Number of SNPs per chromosome in each reference panel

Chromosome	Number of SNPs in each reference panel		
	SABE	1KGP3	SABE+1KGP3
1	3,996,540	6,191,833	7,939,598
2	4,406,141	6,790,551	8,726,263
3	3,690,698	5,641,493	7,257,393
4	3,596,780	5,477,810	7,025,900
5	3,325,574	5,115,036	6,553,929
6	3,174,612	4,863,337	6,218,835
7	2,945,373	4,511,408	5,792,582
8	2,898,843	4,425,449	5,683,312
9	2,204,350	3,384,360	4,346,771
10	2,514,657	3,874,259	4,950,281
11	2,509,089	3,881,791	4,972,826
12	2,421,384	3,745,465	4,800,039
13	1,806,750	2,760,845	3,534,231
14	1,644,170	2,548,903	3,259,739
15	1,484,079	2,301,453	2,949,517
16	1,655,523	2,548,920	3,289,287
17	1,427,164	2,209,149	2,855,082
18	1,432,958	2,189,529	2,800,626
19	1,106,201	1,738,824	2,237,376
20	1,180,936	1,817,492	2,329,578
21	664,678	1,045,269	1,324,116
22	679,009	1,059,079	1,357,134
TOTAL	50,765,509	78,229,219	100,204,415

Supplementary Table 11. Comparison between target haplotype phase inferences with different reference haplotypes using the number of imputed SNPs for chromosomes 15, 17, 20, and 22. Target 2.5M EPIGEN

Imputation Reference Panel	SABE		1KGP3		SABE+1KGP3	
Number of variants	Total	info score ≥ 0.8	Total	info score ≥ 0.8	Total	info score ≥ 0.8
Chr 15	1,481,369	600,332	2,297,258	799,440	2,943,434	951,917
Chr 17	1,424,402	512,055	2,204,724	738,586	2,849,458	866,547
Chr 20	1,180,618	417,420	1,816,925	615,816	2,328,821	703,545
Chr 22	676,922	229,932	1,049,542	351,164	1,345,756	402,144

Supplementary Table 12. Comparison between target haplotype phase inferences with different reference haplotypes using the number of imputed SNPs for chromosomes 15, 17, 20, and 22. Target 2.5M SALVADOR

Imputation Reference Panel	SABE		1KGP3		SABE+1KGP3	
Number of variants	Total	info score ≥ 0.8	Total	info score ≥ 0.8	Total	info score ≥ 0.8
Chr 15	1,481,369	605,791	2,297,258	799,308	2,943,434	921,805
Chr 17	1,424,402	519,213	2,204,724	740,926	2,849,458	851,122
Chr 20	1,180,618	423,629	1,816,925	616,333	2,328,821	691,770
Chr 22	676,922	234,225	1,049,542	352,320	1,345,756	395,895

Supplementary Table 13. omparison between target haplotype phase inferences with different reference haplotypes using the number of imputed SNPs for chromosomes 15, 17, 20, and 22. Target 2.5M PELOTAS

Imputation Reference Panel	SABE		1KGP3		SABE+1KGP3	
Number of variants	Total	info score ≥ 0.8	Total	info score ≥ 0.8	Total	info score ≥ 0.8
Chr 15	1,481,369	594,208	2,297,258	763,178	2,943,434	898,979
Chr 17	1,424,402	509,774	2,204,724	711,297	2,849,458	826,780
Chr 20	1,180,618	414,600	1,816,925	594,121	2,328,821	673,256
Chr 22	676,922	228,107	1,049,542	339,416	1,345,756	384,628

Supplementary Table 14. Comparison between target haplotype phase inferences with different reference haplotypes using the number of imputed SNPs for chromosomes 15, 17, 20, and 22. Target 2.5M BAMBUI

Imputation Reference Panel	SABE		1KGP3		SABE+1KGP3	
Number of variants	Total	info score ≥ 0.8	Total	info score ≥ 0.8	Total	info score ≥ 0.8
Chr 15	1,481,369	573,646	2,297,258	692,257	2,943,434	803,886
Chr 17	1,424,402	495,561	2,204,724	648,734	2,849,458	746,572
Chr 20	1,180,618	403,534	1,816,925	541,084	2,328,821	605,172
Chr 22	676,922	224,813	1,049,542	314,774	1,345,756	354,075

Supplementary Table 15. Comparison between target haplotype phase inferences with different reference haplotypes using the number of imputed SNPs for chromosomes 15, 17, 20, and 22. Target 2.5M Admixed from Peru

Imputation Reference Panel	SABE		1KGP3		SABE+1KGP3	
Number of variants	Total	info score ≥ 0.8	Total	info score ≥ 0.8	Total	info score ≥ 0.8
Chr 15	1,481,369	434,099	2,297,258	533,686	2,943,434	574,401
Chr 17	1,424,518	383,032	2,204,724	495,656	2,849,458	535,641
Chr20	1,180,618	319,760	1,816,925	413,588	2,328,821	442,458
Chr22	676,922	176,627	1,049,542	238,874	1,345,756	255,402

Supplementary Table 16. Comparison between target haplotype phase inferences with different reference haplotypes using the number of imputed SNPs for chromosomes 15, 17, 20, and 22. Target 2.5M Admixed from Guatemala

Imputation Reference Panel	SABE		1KGP3		SABE+1KGP3	
Number of variants	Total	info score ≥ 0.8	Total	info score ≥ 0.8	Total	info score ≥ 0.8
Chr 15	1,481365	369,008	2,297,258	472,194	2,676,512	464,629
Chr 17	1,424,518	316,207	2,204,724	441,305	2,849,458	480,433
Chr20	1,180,618	260,163	1,609,925	319,676	2,274,965	381,225
Chr22	676,922	143,214	1,049,542	210,215	1,345,756	226,126

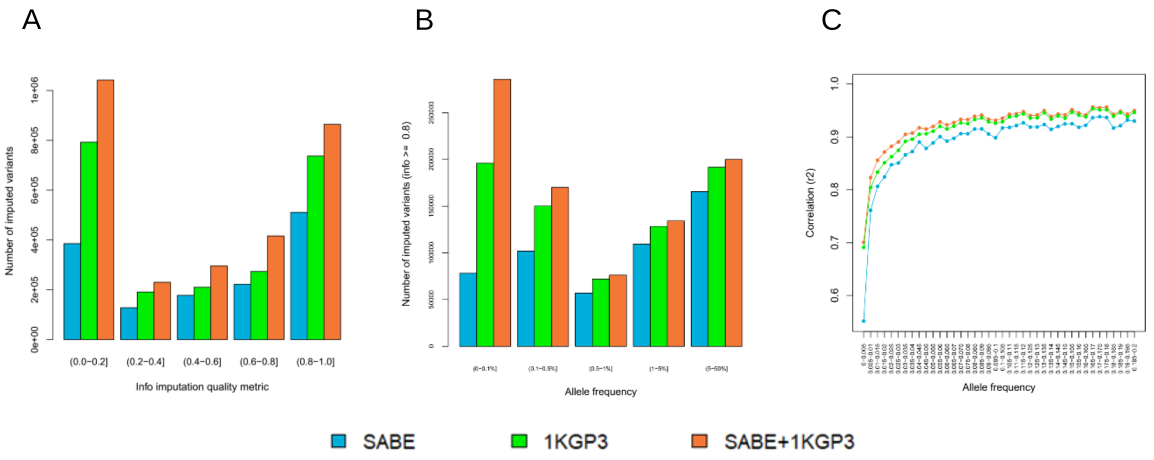

Supplementary Figure 14. Comparison of imputation performance of SABE, 1KGP3, and SABE+1KGP3 reference panels using the Omni 2.5M array data for 6,487 Brazilians from **EPIGEN for chromosome 17** as target panel. **A.** The total number of imputed variants across different classes of the info score quality metric. **B.** The total number of imputed variants with info score ≥ 0.8 across the allele frequency spectrum. **C.** Improvement in imputation accuracy as a function of MAF for the target dataset after imputation (MAF from 0 to 0.2, bin sizes of 0.005).

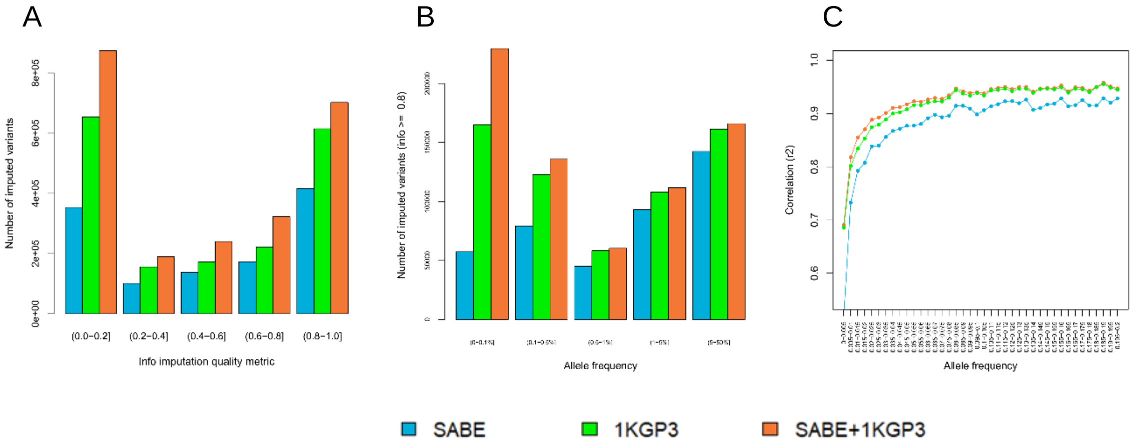

Supplementary Figure 15. Comparison of imputation performance of SABE, 1KGP3, and SABE+1KGP3 reference panels using the Omni 2.5M array data for 6,487 Brazilians from **EPIGEN for chromosome 20** as target panel. **A.** The total number of imputed variants across different classes of the info score quality metric. **B.** The total number of imputed variants with info score ≥ 0.8 across the allele frequency spectrum. **C.** Improvement in imputation accuracy as a function of MAF for the target dataset after imputation (MAF from 0 to 0.2, bin sizes of 0.005).

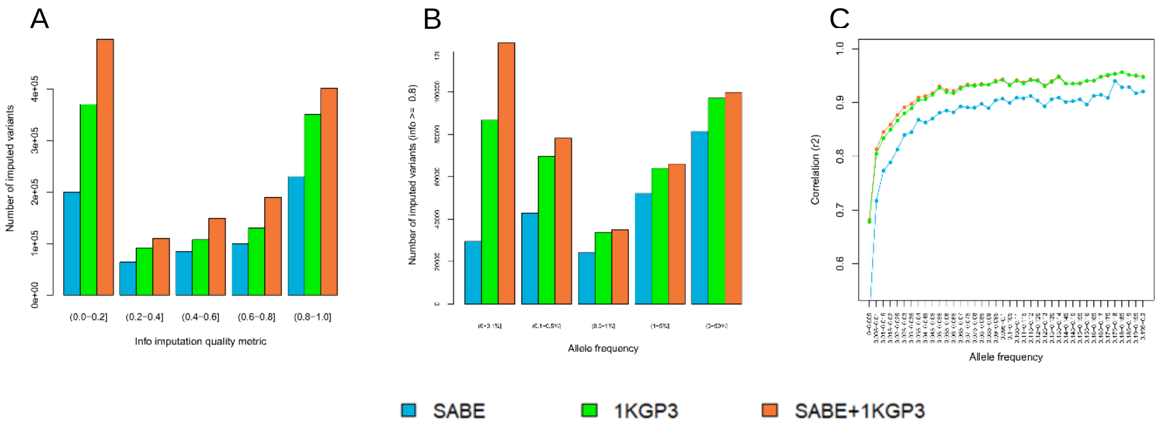

Supplementary Figure 16. Comparison of imputation performance of SABE, 1KGP3, and SABE+1KGP3 reference panels using the Omni 2.5M array data for 6,487 Brazilians from **EPIGEN for chromosome 22** as target panel. **A.** The total number of imputed variants across different classes of the info score quality metric. **B.** The total number of imputed variants with info score ≥ 0.8 across the allele frequency spectrum. **C.** Improvement in imputation accuracy as a function of MAF for the target dataset after imputation (MAF from 0 to 0.2, bin sizes of 0.005).

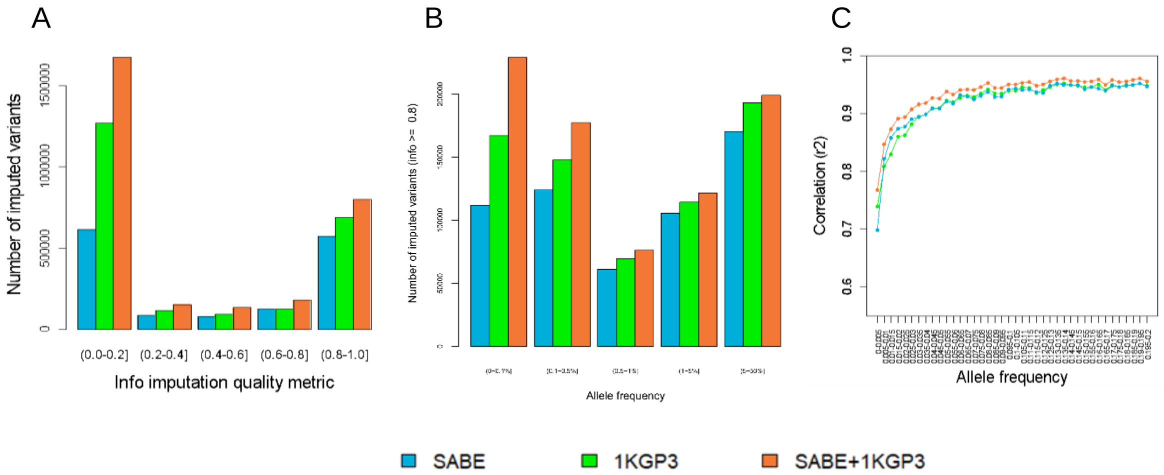

Supplementary Figure 17. Comparison of imputation performance of SABE, 1KGP3, and SABE+1KGP3 reference panels using the Omni 2.5M array data for 1,442 Brazilians from **BAMBUÍ for chromosome 15** as target panel. **A.** The total number of imputed variants across different classes of the info score quality metric. **B.** The total number of imputed variants with info score ≥ 0.8 across the allele frequency spectrum. **C.** Improvement in imputation accuracy as a function of MAF for the target dataset after imputation (MAF from 0 to 0.2, bin sizes of 0.005).

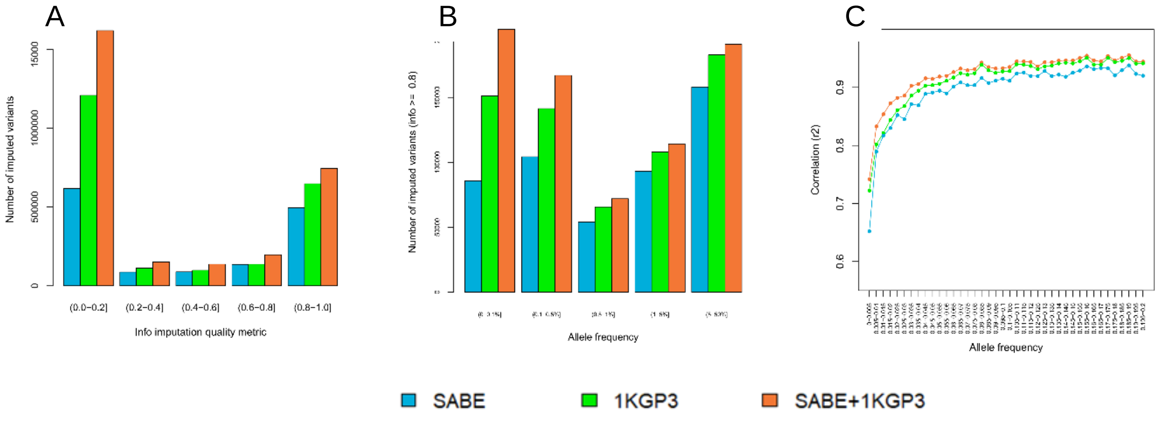

Supplementary Figure 18. Comparison of imputation performance of SABE, 1KGP3, and SABE+1KGP3 reference panels using the Omni 2.5M array data for 1,442 Brazilians from **BAMBUÍ for chromosome 17** as target panel. **A.** The total number of imputed variants across different classes of the info score quality metric. **B.** The total number of imputed variants with info score ≥ 0.8 across the allele frequency spectrum. **C.** Improvement in imputation accuracy as a function of MAF for the target dataset after imputation (MAF from 0 to 0.2, bin sizes of 0.005).

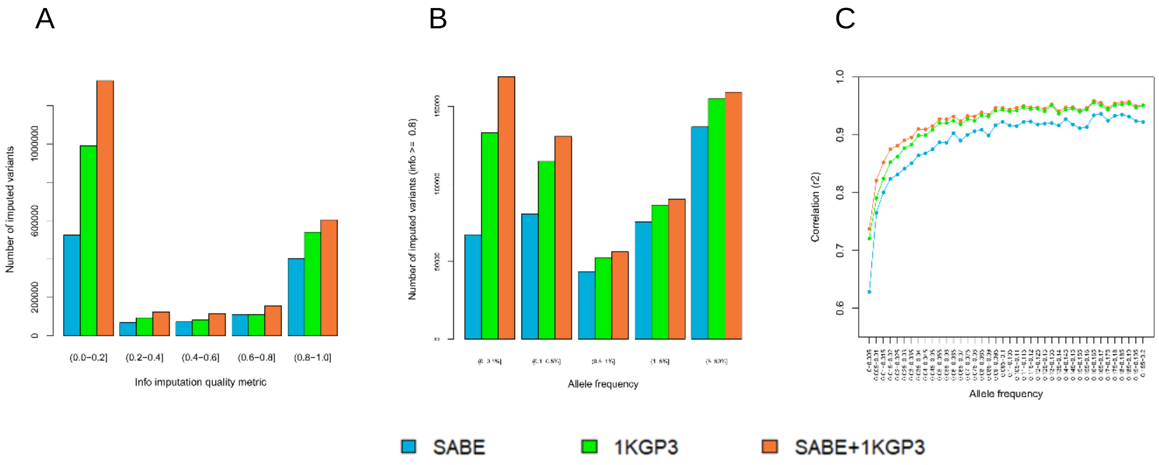

Supplementary Figure 19. Comparison of imputation performance of SABE, 1KGP3, and SABE+1KGP3 reference panels using the Omni 2.5M array data for 1,442 Brazilians from **BAMBUÍ for chromosome 20** as target panel. **A.** The total number of imputed variants across different classes of the info score quality metric. **B.** The total number of imputed variants with info score ≥ 0.8 across the allele frequency spectrum. **C.** Improvement in imputation accuracy as a function of MAF for the target dataset after imputation (MAF from 0 to 0.2, bin sizes of 0.005).

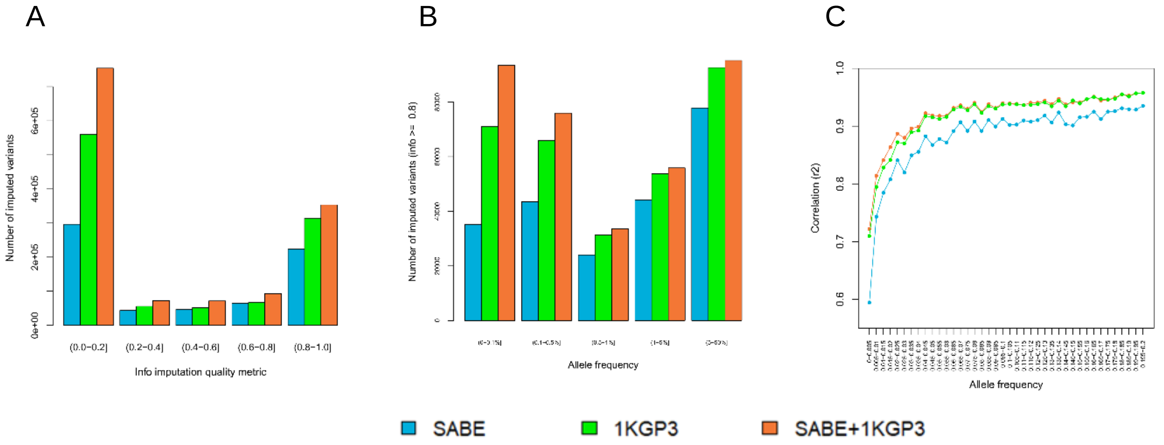

Supplementary Figure 20. Comparison of imputation performance of SABE, 1KGP3, and SABE+1KGP3 reference panels using the Omni 2.5M array data for 1,442 Brazilians from **BAMBUÍ for chromosome 22** as target panel. **A.** The total number of imputed variants across different classes of the info score quality metric. **B.** The total number of imputed variants with info score ≥ 0.8 across the allele frequency spectrum. **C.** Improvement in imputation accuracy as a function of MAF for the target dataset after imputation (MAF from 0 to 0.2, bin sizes of 0.005).

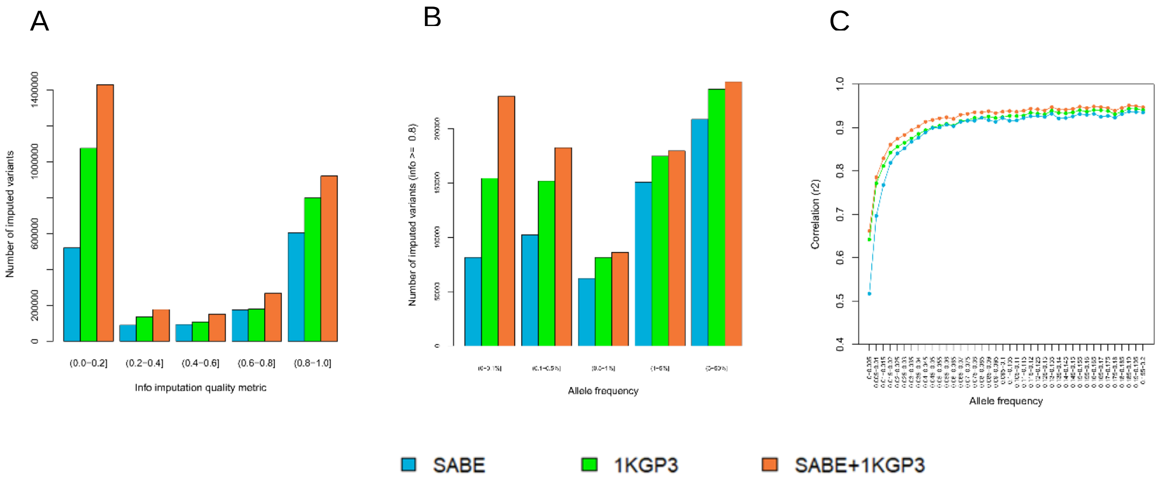

Supplementary Figure 21. Comparison of imputation performance of SABE, 1KGP3, and SABE+1KGP3 reference panels using the Omni 2.5M array data for 1,309 Brazilians from **SALVADOR for chromosome 15** as target panel. **A.** The total number of imputed variants across different classes of the info score quality metric. **B.** The total number of imputed variants with info score ≥ 0.8 across the allele frequency spectrum. **C.** Improvement in imputation accuracy as a function of MAF for the target dataset after imputation (MAF from 0 to 0.2, bin sizes of 0.005).

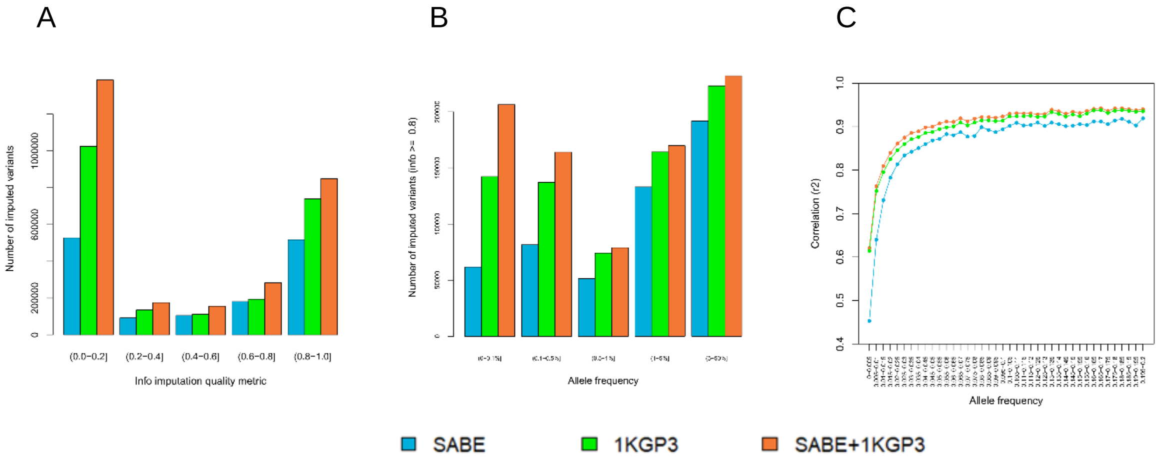

Supplementary Figure 22. Comparison of imputation performance of SABE, 1KGP3, and SABE+1KGP3 reference panels using the Omni 2.5M array data for 1,309 Brazilians from **SALVADOR for chromosome 17** as target panel. **A.** The total number of imputed variants across different classes of the info score quality metric. **B.** The total number of imputed variants with info score ≥ 0.8 across the allele frequency spectrum. **C.** Improvement in imputation accuracy as a function of MAF for the target dataset after imputation (MAF from 0 to 0.2, bin sizes of 0.005).

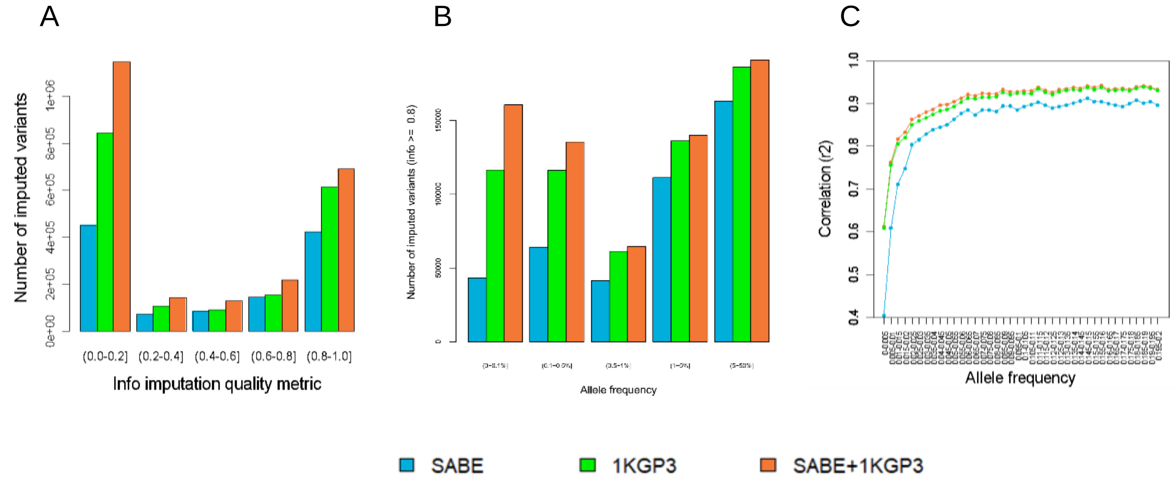

Supplementary Figure 23. Comparison of imputation performance of SABE, 1KGP3, and SABE+1KGP3 reference panels using the Omni 2.5M array data for 1,309 Brazilians from **SALVADOR for chromosome 20** as target panel. **A.** The total number of imputed variants across different classes of the info score quality metric. **B.** The total number of imputed variants with info score ≥ 0.8 across the allele frequency spectrum. **C.** Improvement in imputation accuracy as a function of MAF for the target dataset after imputation (MAF from 0 to 0.2, bin sizes of 0.005).

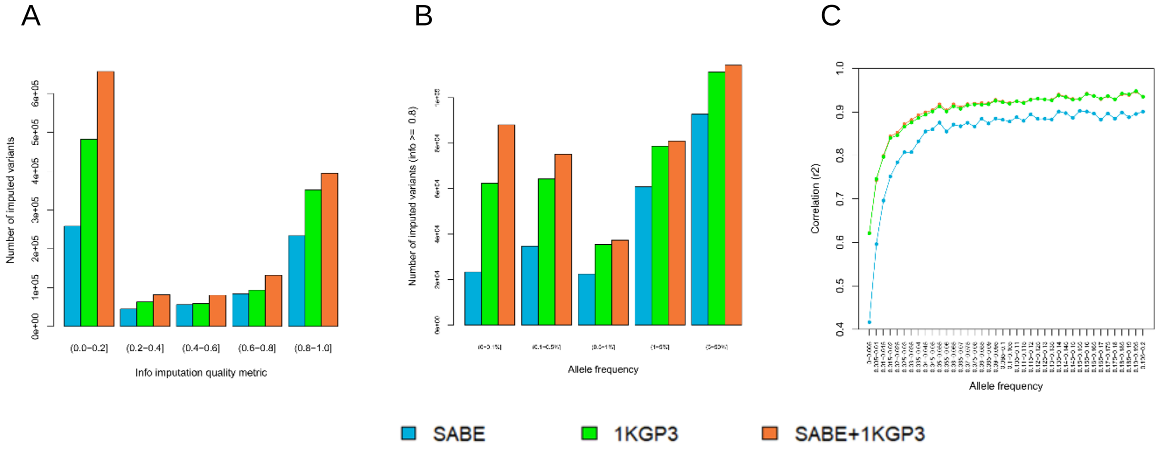

Supplementary Figure 24. Comparison of imputation performance of SABE, 1KGP3, and SABE+1KGP3 reference panels using the Omni 2.5M array data for 1,309 Brazilians from **SALVADOR for chromosome 22** as target panel. **A.** The total number of imputed variants across different classes of the info score quality metric. **B.** The total number of imputed variants with info score ≥ 0.8 across the allele frequency spectrum. **C.** Improvement in imputation accuracy as a function of MAF for the target dataset after imputation (MAF from 0 to 0.2, bin sizes of 0.005).

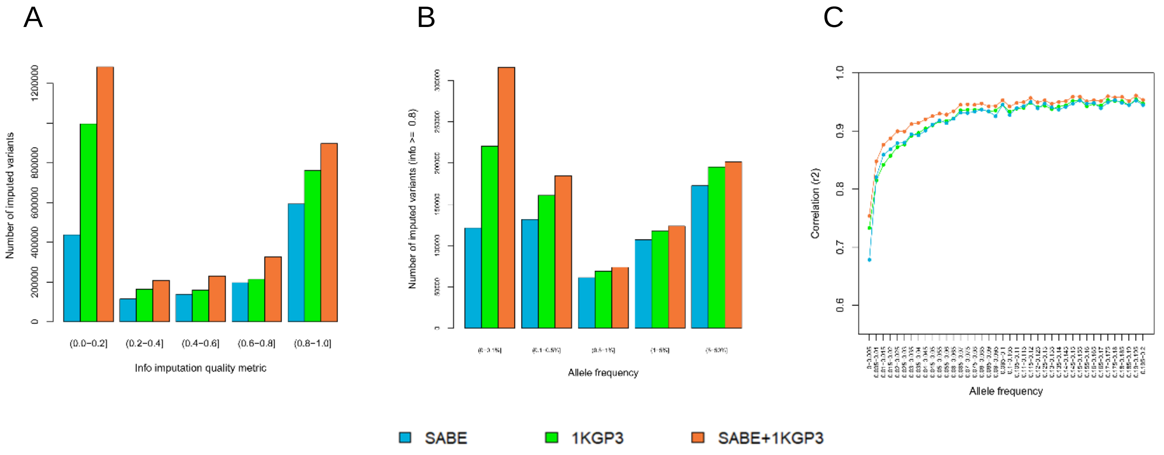

Supplementary Figure 25. Comparison of imputation performance of SABE, 1KGP3, and SABE+1KGP3 reference panels using the Omni 2.5M array data for 3,736 Brazilians from **PELOTAS for chromosome 15** as target panel. **A.** The total number of imputed variants across different classes of the info score quality metric. **B.** The total number of imputed variants with info score ≥ 0.8 across the allele frequency spectrum. **C.**

Improvement in imputation accuracy as a function of MAF for the target dataset after imputation (MAF from 0 to 0.2, bin sizes of 0.005).

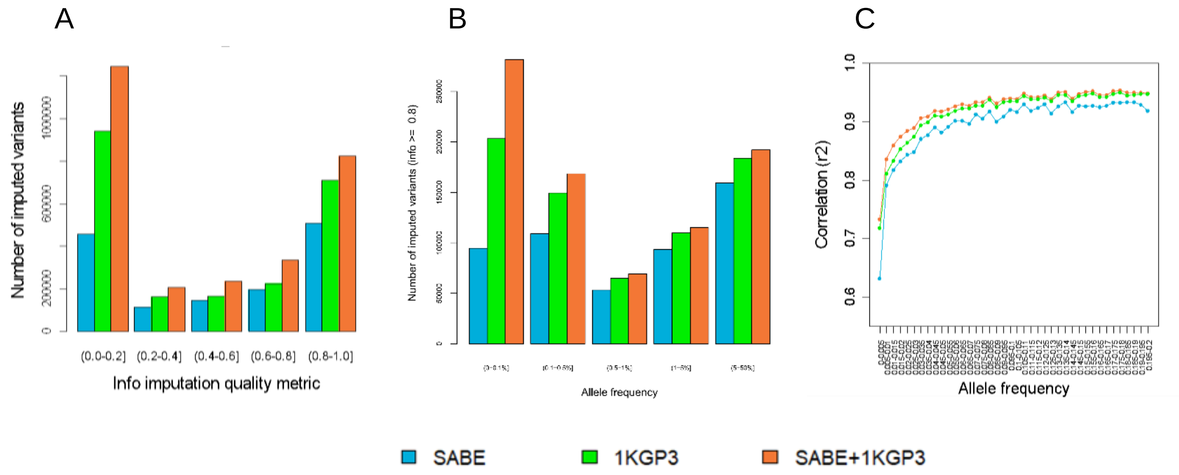

Supplementary Figure 26. Comparison of imputation performance of SABE, 1KGP3, and SABE+1KGP3 reference panels using the Omni 2.5M array data for 3,736 Brazilians from **PELOTAS for chromosome 17** as target panel. **A.** The total number of imputed variants across different classes of the info score quality metric. **B.** The total number of imputed variants with info score ≥ 0.8 across the allele frequency spectrum. **C.** Improvement in imputation accuracy as a function of MAF for the target dataset after imputation (MAF from 0 to 0.2, bin sizes of 0.005).

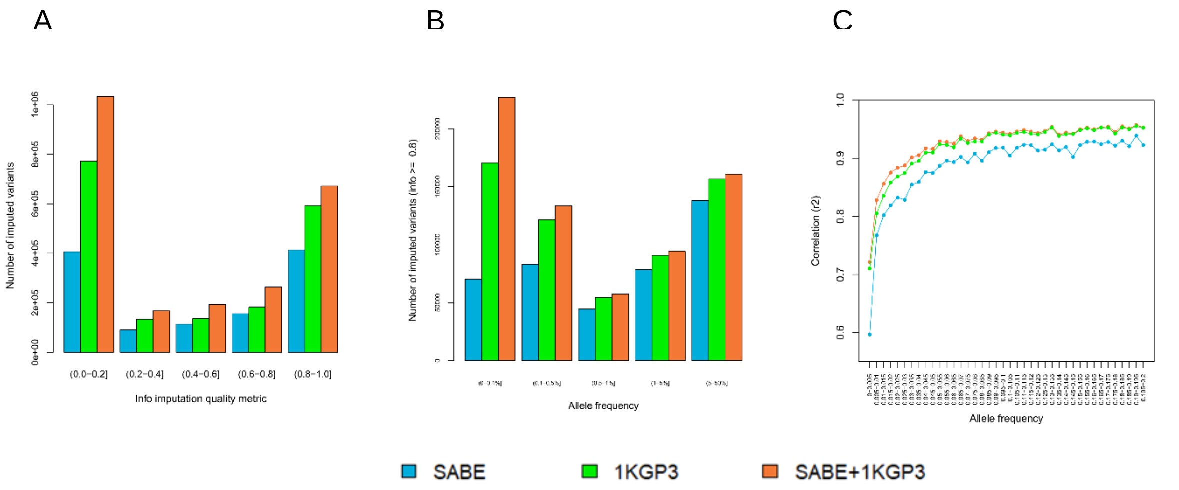

Supplementary Figure 27. Comparison of imputation performance of SABE, 1KGP3, and SABE+1KGP3

reference panels using the Omni 2.5M array data for 3,736 Brazilians from **PELOTAS for chromosome 20** as target panel. **A.** The total number of imputed variants across different classes of the info score quality metric. **B.** The total number of imputed variants with info score ≥ 0.8 across the allele frequency spectrum. **C.** Improvement in imputation accuracy as a function of MAF for the target dataset after imputation (MAF from 0 to 0.2, bin sizes of 0.005).

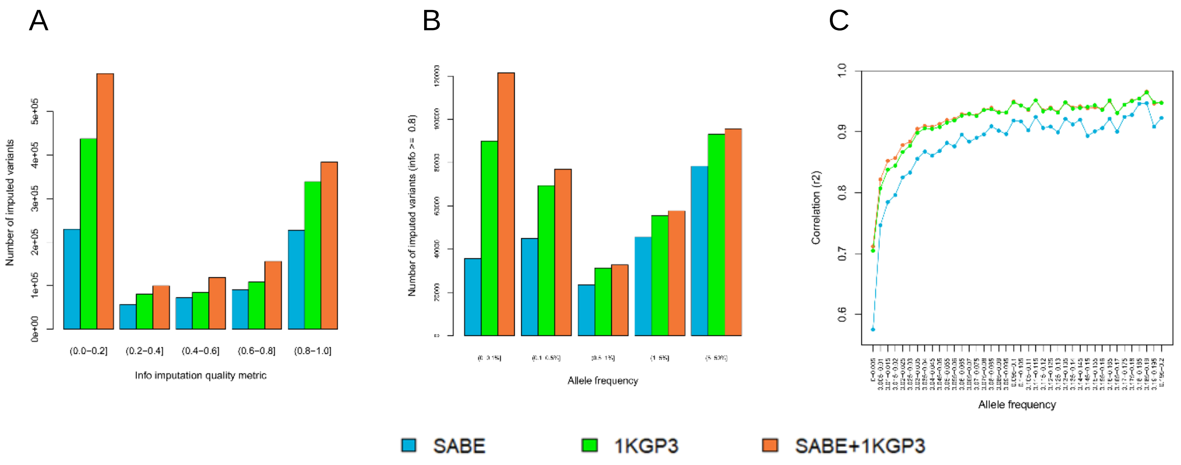

Supplementary Figure 28. Comparison of imputation performance of SABE, 1KGP3, and SABE+1KGP3 reference panels using the Omni 2.5M array data for 3,736 Brazilians from **PELOTAS for chromosome 22** as target panel. **A.** The total number of imputed variants across different classes of the info score quality metric. **B.** The total number of imputed variants with info score ≥ 0.8 across the allele frequency spectrum. **C.** Improvement in imputation accuracy as a function of MAF for the target dataset after imputation (MAF from 0 to 0.2, bin sizes of 0.005).

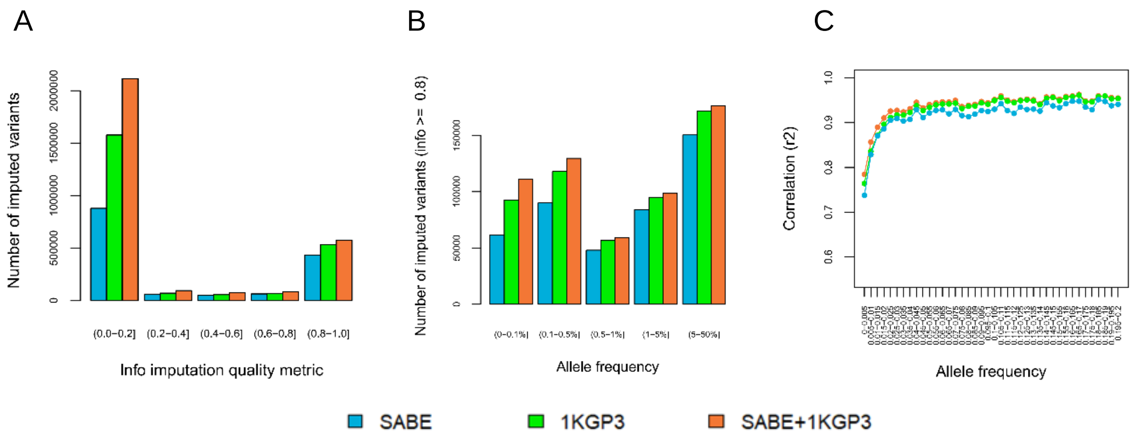

Supplementary Figure 29. Comparison of imputation performance of SABE, 1KGP3, and SABE+1KGP3 reference panels using the Omni 2.5M array data for 391 Mestizos from **Peru for chromosome 15** as target panel. **A.** The total number of imputed variants across different classes of the info score quality metric. **B.** The total number of imputed variants with info score ≥ 0.8 across the allele frequency spectrum. **C.** Improvement in imputation accuracy as a function of MAF for the target dataset after imputation (MAF from 0 to 0.2, bin sizes of 0.005).

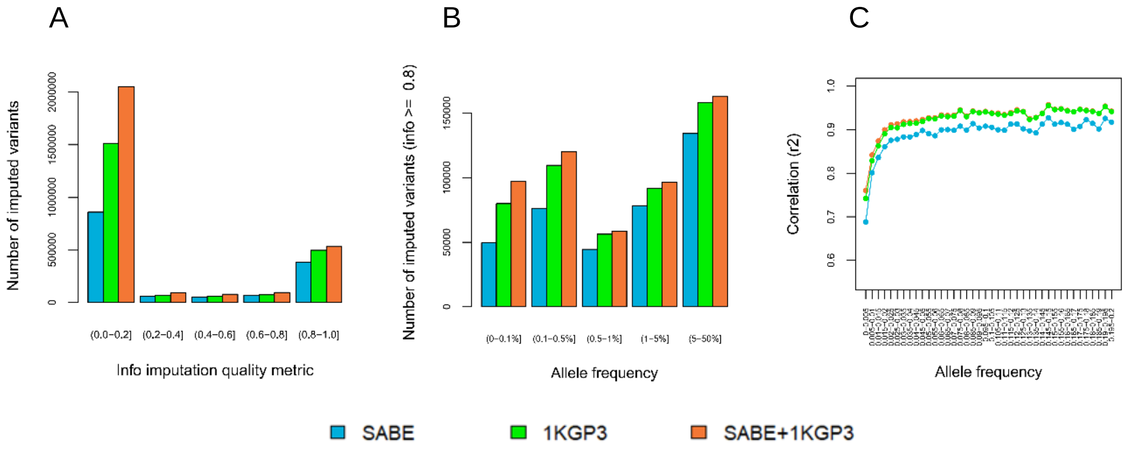

Supplementary Figure 30. Comparison of imputation performance of SABE, 1KGP3, and SABE+1KGP3 reference panels using the Omni 2.5M array data for 391 Mestizos from **Peru for chromosome 17** as target panel. **A.** The total number of imputed variants across different classes of the info score quality metric. **B.** The total number of imputed variants with info score ≥ 0.8 across the allele frequency spectrum. **C.** Improvement in imputation accuracy as a function of MAF for the target dataset after imputation (MAF from 0 to 0.2, bin sizes of 0.005).

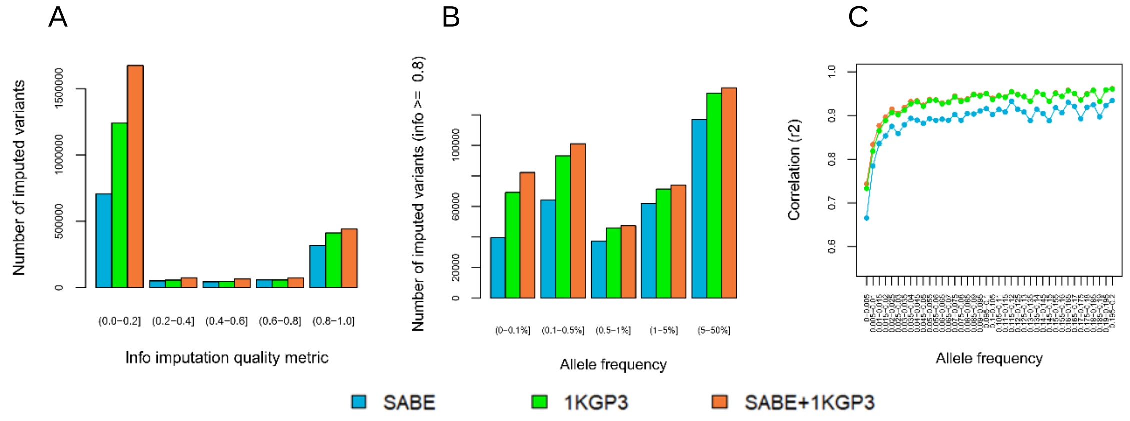

Supplementary Figure 31. Comparison of imputation performance of SABE, 1KGP3, and SABE+1KGP3 reference panels using the Omni 2.5M array data for 391 Mestizos from **Peru for chromosome 20** as target panel. **A.** The total number of imputed variants across different classes of the info score quality metric. **B.** The total number of imputed variants with info score ≥ 0.8 across the allele frequency spectrum. **C.** Improvement in imputation accuracy as a function of MAF for the target dataset after imputation (MAF from 0 to 0.2, bin sizes of 0.005).

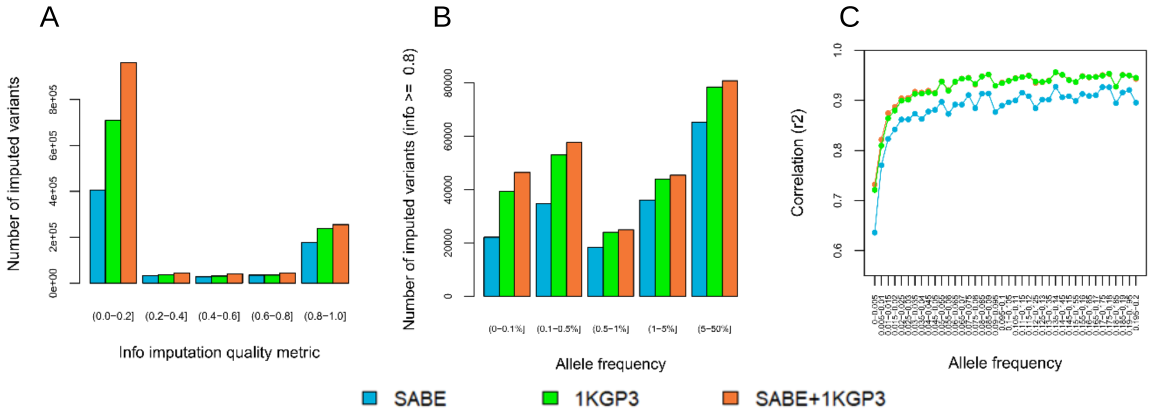

Supplementary Figure 32. Comparison of imputation performance of SABE, 1KGP3, and SABE+1KGP3 reference panels using the Omni 2.5M array data for 391 Mestizos from **Peru for chromosome 22** as target panel. **A.** The total number of imputed variants across different classes of the info score quality metric. **B.** The total number of imputed variants with info score ≥ 0.8 across the allele frequency spectrum. **C.** Improvement in imputation accuracy as a function of MAF for the target dataset after imputation (MAF from 0 to 0.2, bin sizes of 0.005).

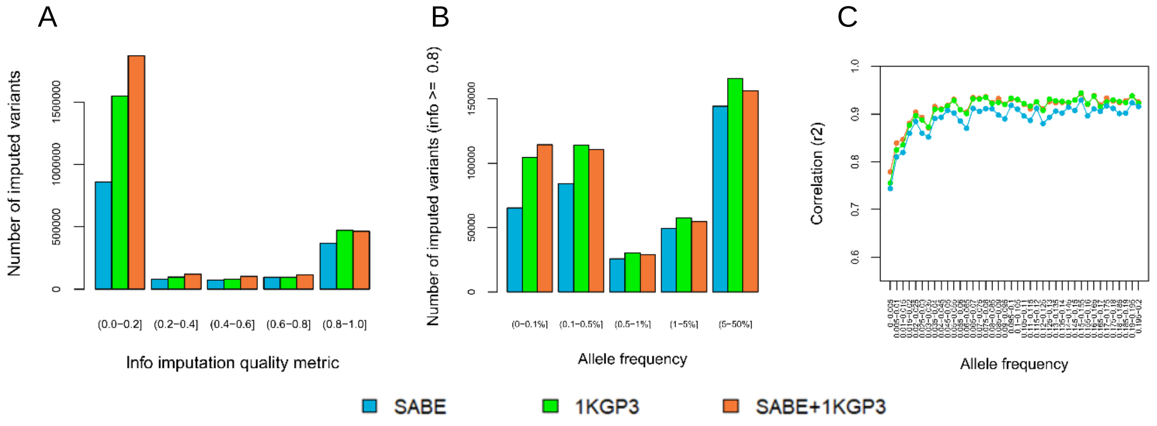

Supplementary Figure 33. Comparison of imputation performance of SABE, 1KGP3, and SABE+1KGP3 reference panels using the oncoarray data for 640 individuals from **Guatemala for chromosome 15** as target panel. **A.** The total number of imputed variants across different classes of the info score quality metric. **B.** The total number of imputed variants with info score ≥ 0.8 across the allele frequency spectrum. **C.** Improvement in imputation accuracy as a function of MAF for the target dataset after imputation (MAF from 0 to 0.2, bin sizes of 0.005).

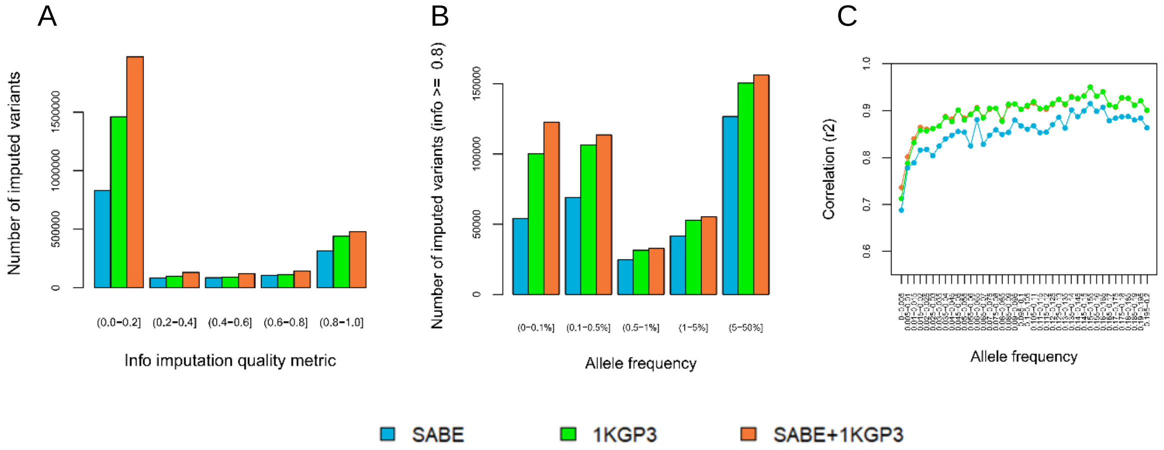

Supplementary Figure 34. Comparison of imputation performance of SABE, 1KGP3, and SABE+1KGP3 reference panels using the oncoarray data for 640 individuals from **Guatemala for chromosome 17** as target panel. **A.** The total number of imputed variants across different classes of the info score quality metric. **B.** The total number of imputed variants with info score ≥ 0.8 across the allele frequency spectrum. **C.** Improvement in imputation accuracy as a function of MAF for the target dataset after imputation (MAF from 0 to 0.2, bin sizes of 0.005).

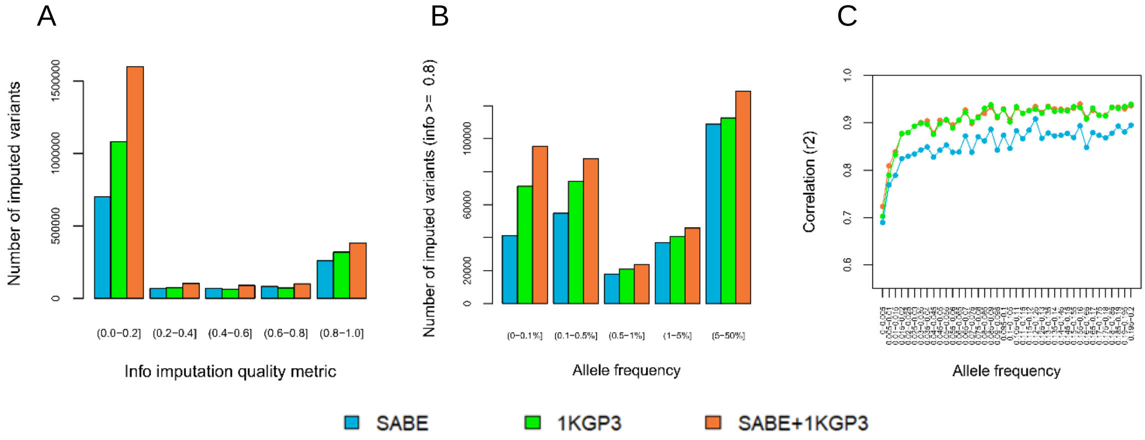

Supplementary Figure 35. Comparison of imputation performance of SABE, 1KGP3, and SABE+1KGP3 reference panels using the oncoarray data for 640 individuals from **Guatemala for chromosome 20** as target panel. **A.** The total number of imputed variants across different classes of the info score quality metric. **B.** The total number of imputed variants with info score ≥ 0.8 across the allele frequency spectrum. **C.** Improvement in imputation accuracy as a function of MAF for the target dataset after imputation (MAF from 0 to 0.2, bin sizes of 0.005).

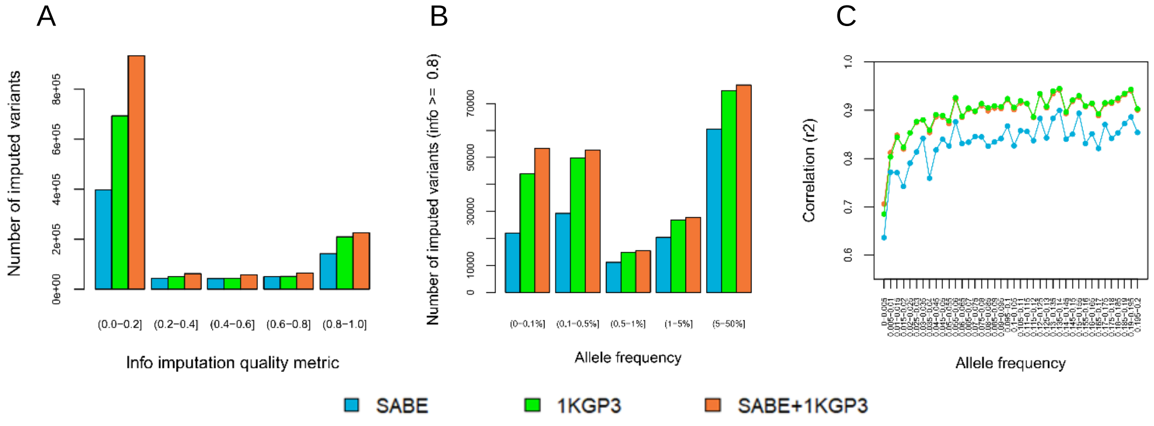

Supplementary Figure 36. Comparison of imputation performance of SABE, 1KGP3, and SABE+1KGP3 reference panels using the oncoarray data for 640 individuals from **Guatemala for chromosome 22** as target panel. **A.** The total number of imputed variants across different classes of the info score quality metric. **B.** The total number of imputed variants with info score ≥ 0.8 across the allele frequency spectrum. **C.** Improvement in imputation accuracy as a function of MAF for the target dataset after imputation (MAF from 0 to 0.2, bin sizes of 0.005).

Supplementary Note 8: HLA

The *hla-mapper* software was designed to optimize read mapping in HLA genes by comparing each read to a database of known HLA sequences and calculating where each read should be mapped or considered ambiguous[10]. This step is essential to get accurate genotype calls in the SNP-level for HLA genes. We used an updated version of this software, version 4, with support to intergenic sequences and faster processing WGS data.

After the map optimization, we used GATK HaplotypeCaller version 4.1.7 to call genotypes in the genome confidence model (GVCF), concatenating all samples together in a VCF file using GenotypeGVCFs. HaplotypeCaller can detect both SNPs and indels. For variant refinement, we noticed that the VQSR-AS approach does not produce reliable results for the HLA region by observing the filtered variants in samples with known HLA alleles. Because of that, we used a different approach for variant refinement and selection for HLA genes. We used a local program (vcfx) that introduces missing alleles in unbalanced genotypes (vcfx checked) and genotypes with a low likelihood (vcfx checkpl), and annotate each variant with some quantitative parameters such as the number of balanced heterozygous variants (when both alleles present similar depth of coverage), number of homozygous samples, the proportion of missing alleles, and others (vcfx evidence). Each variant that has not been approved by the vcfx evidence algorithm was evaluated manually. Each variant was annotated using the dbSNP dataset version 151.

We inferred haplotypes combining two computational strategies. First, we used GATK ReadBackedPhasing (RBP) to detect the phase between close heterozygous variants. The minimal Phase Quality Threshold was set to 500 (25x the default value). This procedure produced phase sets, i.e., blocks of known phases, but unphased among each other. RBP does not consider Multi-allelic variants, indels, and missing alleles. The second step consisted of inferring the full haplotypes using probabilistic models, but considering the phase sets detected by RBP. For that, we used an in-house software named phasex (available upon request), that uses Shapeit4[11] to phase bi-allelic variants considering the RBP's phase sets, in 20 independent runs with different seeds and using a single core per independent run, comparing the results afterward. The independent runs can be parallelized according to the number of cores on the computer. We preserved the haplotypes of all samples in which the same pair of haplotypes was observed in at least 18 runs ($P > 0.9$), passing these haplotypes forward to the next round. Iterations were performed until the number of samples with $P > 0.9$ no longer increased. Then, the haplotypes that have been detected are passed forward to the next step. In this next step, we use Beagle 4.1[12] to infer the final set of haplotypes, now including the multi-allelic variants. The same iteration procedure is used, with 20 independent runs and fixing haplotypes with $P > 0.9$. For each sample, we considered the haplotype with $P > 0.7$ after the last Beagle run. Shapeit4 and Beagle 4.1 also imputed the bi-allelic and multi-allelic missing alleles, which were introduced by the vcfx approach. It should be noted that we removed all singletons before the haplotyping procedure. This step is necessary because singletons are ambiguous by definition and impair haplotyping performance. Singletons were automatically inserted in the final VCF file using a local Perl script as unphased or phased, depending on the singleton's RBP status.

To generate complete sequences and CDS sequences (only exons) for each HLA gene and each sample, we used the vcfx fasta and vcfx transcript functions. We also compared our sequences with the ones described in the IPD-IMGT/HLA Database version 3.4.0[13] using a local Perl script, identifying whether they were identical or not to known sequences in the database. The CDS sequences were translated into full-length proteins using EMBOSS transeq and named according to the IPD-IMGT/HLA database. Allele, genotype, and haplotype frequencies were calculated by direct counting. Variants were annotated using SNPeff[14]. Results on HLA haplotypes, distribution of known and previously not described, frequencies distribution and annotation are summarized in Supplementary Figure 37.

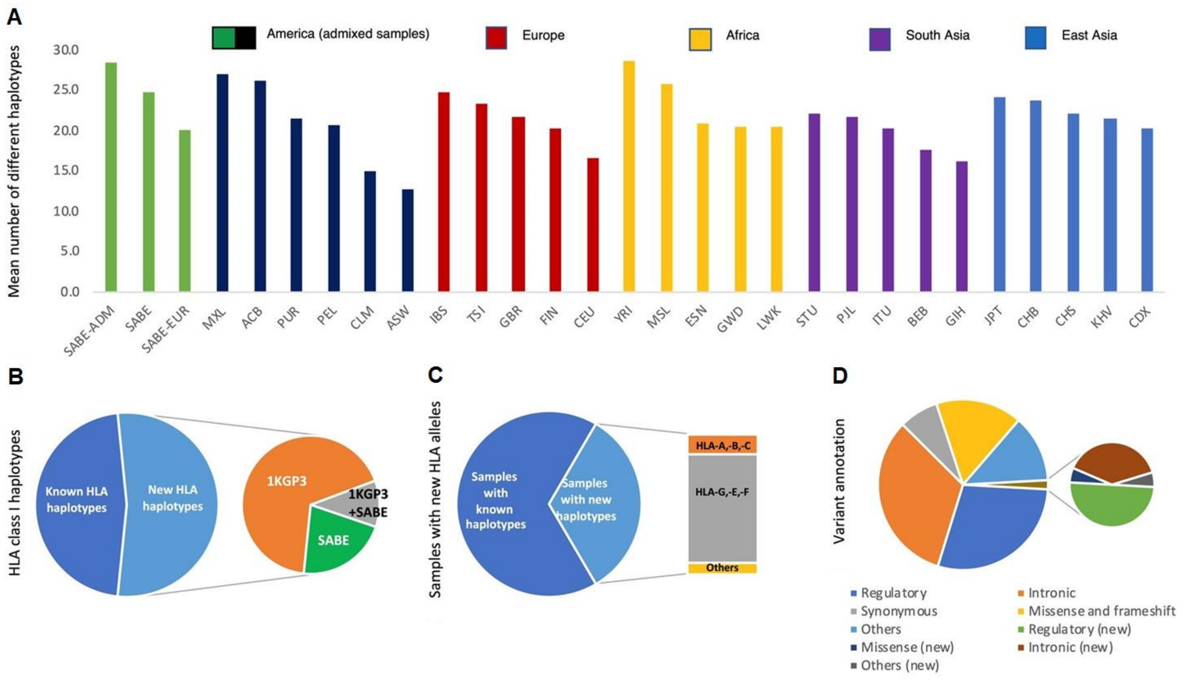

Supplementary Figure 37. HLA polymorphism in the SABE cohort. A. The average number of different HLA haplotypes observed in 10,000 resamplings of 50 individuals, considering genes *HLA-A*, *HLA-B*, *HLA-C*, *HLA-E*, *HLA-F*, and *HLA-G*. SABE: all samples from Brazil; SABE-ADM: samples with at least 30% of European and African ancestry; SABE-EUR: samples with 100% European ancestry. **B.** Distribution of known and new HLA haplotypes in the SABE+1KGP3 combined dataset compared to the IPD-IMGT/HLA database. We detected 682 new haplotypes (53.2% of all observed haplotypes), 21% exclusively in SABE (green). The cumulative frequency of these new haplotypes is 4.43%. **C.** Frequency of individuals in SABE with a new HLA allele (33%, light blue), and where these new alleles were detected. Most of the novelty was observed for non-classical HLA class I alleles (gray). **D.** HLA variant annotation and the proportion of new variants detected in the SABE cohort according to dbSNP.

Supplementary Note 9: HLA Imputation

Supplementary Table 17. Number of samples and alleles in each reference panel (1KGP3, SABE and SABE+1KGP3) and the out-of-bag accuracy for the HLA imputation models with 2 fields resolution.

Locus	Samples in the reference panel	N alleles in the reference panel (2 fields resolution)	Average Out-of-bag Accuracy
		1KGP3	
HLA-A	2503	82	91.33% ± 0.79%
HLA-B	2498	154	86.36% ± 0.87%
HLA-C	2503	63	97.31% ± 0.41%
		SABE	
HLA-A	1171	68	92.47% ± 0.81%
HLA-B	1171	107	85.61% ± 1.12%
HLA-C	1171	45	97.72% ± 0.53%
		SABE + 1KGP3	
HLA-A	3674	102	90.28% ± 0.54%
HLA-B	3669	176	86.33% ± 0.62%
HLA-C	3674	74	97.58% ± 0.29%

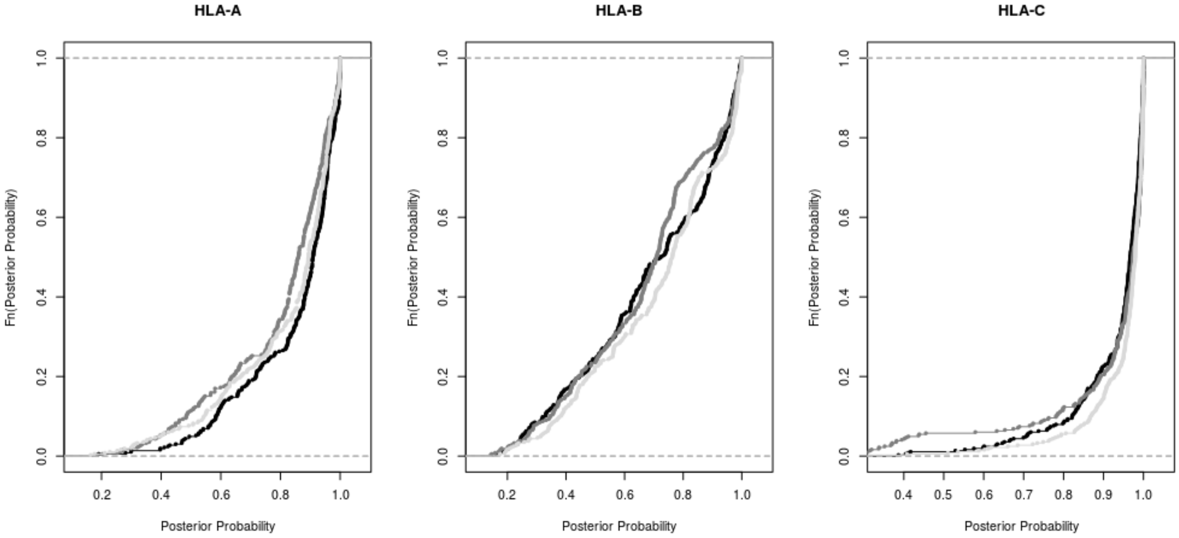

Supplementary Figure 14. Empirical cumulative distribution function (ECDF) of posterior probabilities for the HLA imputation models: 1KGP3 (black), SABE (dark gray) and SABE+1KGP3 (light gray).

Supplementary References

1 Karczewski, K. J. *et al.* The mutational constraint spectrum quantified from variation in 141,456 humans. *Nature* **581**, 434-443, doi:10.1038/s41586-020-2308-7 (2020).

2 Borda, V. *et al.* The genetic structure and adaptation of Andean highlanders and Amazonians are influenced by the interplay between geography and culture. *Proc Natl Acad Sci U S A* **117**, 32557-32565, doi:10.1073/pnas.2013773117 (2020).

3 Lebrao, M. L., Duarte, Y. A. O., Santos, J. L. F. & Silva, N. N. D. 10 Years of SABE Study: background, methodology and organization of the study. *Rev Bras Epidemiol* **21Suppl 02**, e180002, doi:10.1590/1980-549720180002.supl.2 (2019).

4 Naslavsky, M. S. *et al.* Exomic variants of an elderly cohort of Brazilians in the ABraOM database. *Hum Mutat* **38**, 751-763, doi:10.1002/humu.23220 (2017).

5 Telenti, A. *et al.* Deep sequencing of 10,000 human genomes. *Proc Natl Acad Sci U S A* **113**, 11901-11906, doi:10.1073/pnas.1613365113 (2016).

6 Van der Auwera, G. A. *et al.* From FastQ data to high confidence variant calls: the Genome Analysis Toolkit best practices pipeline. *Curr Protoc Bioinformatics* **43**, 11 10 11-33, doi:10.1002/0471250953.bi1110s43 (2013).

7 Genomes Project, C. *et al.* A global reference for human genetic variation. *Nature* **526**, 68-74, doi:10.1038/nature15393 (2015).

8 Richards, S. *et al.* Standards and guidelines for the interpretation of sequence variants: a joint consensus recommendation of the American College of Medical Genetics and Genomics and the Association for Molecular Pathology. *Genetics in medicine : official journal of the American College of Medical Genetics* **17**, 405-424, doi:10.1038/gim.2015.30 (2015).

9 Kalia, S. S. *et al.* Recommendations for reporting of secondary findings in clinical exome and genome sequencing, 2016 update (ACMG SF v2.0): a policy statement of the American College of Medical Genetics and Genomics. *Genetics in medicine : official journal of the American College of Medical Genetics* **19**, 249-255, doi:10.1038/gim.2016.190 (2017).

10 Castelli, E. C., Paz, M. A., Souza, A. S., Ramalho, J. & Mendes-Junior, C. T. Hla-mapper: An application to optimize the mapping of HLA sequences produced by massively parallel sequencing procedures. *Hum Immunol* **79**, 678-684, doi:10.1016/j.humimm.2018.06.010 (2018).

11 Delaneau, O., Zagury, J. F., Robinson, M. R., Marchini, J. L. & Dermitzakis, E. T. Accurate, scalable and integrative haplotype estimation. *Nature communications* **10**, 5436, doi:10.1038/s41467-019-13225-y (2019).

12 Browning, S. R. & Browning, B. L. Rapid and accurate haplotype phasing and missing-data inference for whole-genome association studies by use of localized haplotype clustering. *Am J Hum Genet* **81**, 1084-1097, doi:10.1086/521987 (2007).

13 Robinson, J. *et al.* IPD-IMGT/HLA Database. *Nucleic Acids Res* **48**, D948-D955, doi:10.1093/nar/gkz950 (2020).

14 Cingolani, P. *et al.* A program for annotating and predicting the effects of single nucleotide polymorphisms, SnpEff: SNPs in the genome of Drosophila melanogaster strain w1118; iso-2; iso-3. *Fly (Austin)* **6**, 80-92, doi:10.4161/fly.19695 (2012).
