# Supplementary material for: Whole-genome sequencing of 1,171 elderly admixed individuals from Brazil
Source: Nat Commun. 2022 Mar 4;13:1004. doi: 10.1038/s41467-022-28648-3 (PMC8897431; doi:10.1038/s41467-022-28648-3)
Supplement: Supplementary file 3 — Description of Additional Supplementary Files [file 41467_2022_28648_MOESM3_ESM.pdf]

Description of Additional Supplementary Files

File Name: Supplementary Data 1
Description: List of tools and versions applied in the bioinformatics pipeline.

File Name: Supplementary Data 2
Description: List of 4,250 OMIM disease genes used in clinical analyses section containing Approved_Symbol, Gene_Name, MIM_number and Phenotypes.

File Name: Supplementary Data 3
Description: Pathogenic variants in OMIM Disease genes with frequencies above 10% in SABE dataset.

File Name: Supplementary Data 4
Description: Reclassified variants after manual curation in genes with dominant mode of inheritance.

File Name: Supplementary Data 5
Description: Pathogenic variants and categories after manual curation in genes with dominant mode of inheritance.

File Name: Supplementary Data 6
Description: Pathogenic findings on ACMG-59 genes.

File Name: Supplementary Data 7
Description: Variant-based incidence of selected genes associated with recessively inherited disorders.

File Name: Supplementary Data 8
Description: OMIM genes with mobile element insertion events in exonic regions.
