# Supplementary material for: Whole-genome sequencing of 1,171 elderly admixed individuals from Brazil
Source: Nat Commun. 2022 Mar 4;13:1004. doi: 10.1038/s41467-022-28648-3 (PMC8897431; doi:10.1038/s41467-022-28648-3)
Supplement: Supplementary file 12 — Reporting Summary [file 41467_2022_28648_MOESM12_ESM.pdf]

nature research

Corresponding author(s): Michel Satya Naslavsky

Last updated by author(s): Oct 27, 2021

Reporting Summary

Nature Research wishes to improve the reproducibility of the work that we publish. This form provides structure for consistency and transparency in reporting. For further information on Nature Research policies, see our Editorial Policies and the Editorial Policy Checklist.

Statistics

For all statistical analyses, confirm that the following items are present in the figure legend, table legend, main text, or Methods section.

n/a	Confirmed	
☐	☒	The exact sample size (*n*) for each experimental group/condition, given as a discrete number and unit of measurement
☐	☒	A statement on whether measurements were taken from distinct samples or whether the same sample was measured repeatedly
☐	☒	The statistical test(s) used AND whether they are one- or two-sided<br>*Only common tests should be described solely by name; describe more complex techniques in the Methods section.*
☐	☒	A description of all covariates tested
☐	☒	A description of any assumptions or corrections, such as tests of normality and adjustment for multiple comparisons
☐	☒	A full description of the statistical parameters including central tendency (e.g. means) or other basic estimates (e.g. regression coefficient) AND variation (e.g. standard deviation) or associated estimates of uncertainty (e.g. confidence intervals)
☐	☒	For null hypothesis testing, the test statistic (e.g. *F*, *t*, *r*) with confidence intervals, effect sizes, degrees of freedom and *P* value noted<br>*Give P values as exact values whenever suitable.*
☐	☒	For Bayesian analysis, information on the choice of priors and Markov chain Monte Carlo settings
☒	☐	For hierarchical and complex designs, identification of the appropriate level for tests and full reporting of outcomes
☒	☐	Estimates of effect sizes (e.g. Cohen's *d*, Pearson's *r*), indicating how they were calculated

Our web collection on statistics for biologists contains articles on many of the points above.

Software and code

Policy information about availability of computer code

Data collection	No software was used for data collection. Datasets used in analyses were manually accessed.
Data analysis	Pipelines used on this study and described within Methods and Supplementary Information are published and cited accordingly (Methods, Supplementary Table 3). Software used: ISIS Isaac Aligner (v. 2.5.26.13; aligner v. 1.14.02.06), GATK release 3.7, In-house script for splitting multiallelic variants, BCFtools (43983), GATK release 3.7 (GATK-DepthOfCoverage), CEGH-Filter (v.1.2), KING (v.1.4), PC-Relate (GENESIS R/Bioconductor package), verifyBAMID, In-house script for X and Y read counts, ADMIXTURE (v.1.3.0), PLINK (v.1.9), SNPRelate, ANNOVAR, MELT (MELT-SPLIT, MELT-Deletion v.2.1.4), hla-mapper (v.4), GATK HaplotypeCaller (v.4.1.7), VCFx (vcfx checkpl, checkad, and evidence v1.2b), GATK ReadBackedPhasing, HIBAG (v1.4), Megahit assembler, minimap2, bowtie2, 10X Chromium linked-reads, HGDP, HGSVC, SHAPEIT2, extractPIRs, IMPUTE2, CrossMap, plink2 (v2), PCrelate, R (qqman), Haploview.

For manuscripts utilizing custom algorithms or software that are central to the research but not yet described in published literature, software must be made available to editors and reviewers. We strongly encourage code deposition in a community repository (e.g. GitHub). See the Nature Research guidelines for submitting code & software for further information.

Data

Policy information about availability of data

All manuscripts must include a data availability statement. This statement should provide the following information, where applicable:

- Accession codes, unique identifiers, or web links for publicly available datasets
- A list of figures that have associated raw data
- A description of any restrictions on data availability

The publicly available genomic dataset analyzed on this study is aggregated as a cohort and presented as short variants and frequencies deposited at the ABraOM [https://abraom.ib.usp.br], where they can be consulted and downloaded for academic research purposes via direct request at the website. ABraOM does

not issue datasets with DOIs. Variants and frequencies were also submitted to dbSNP (to be published in the b156 release). Like the detection of short variants, class I HLA alleles and annotated mobile element insertions were detected using published software. Their lists of variants and respective frequencies are also available at ABraOM [https://abraom.ib.usp.br]. Imputation panels can be requested to corresponding authors. Individual level sequence datasets (BAM files) and variant calling datasets (gVCF files) have been deposited at the European Genome-phenome Archive (EGA), which is hosted by the EBI and the CRG, under EGA Study accession number EGAS00001005052 [https://ega-archive.org/studies/EGAS00001005052]. Further information about EGA can be found on https://ega-archive.org. All requests shall be made through EGA, to be evaluated and approved by the appointed Data Access Committee (DAC). SABE individual-level phenotypic data are not authorized by IRB to be uploaded to a public repository, although a direct collaboration is possible. Requests for phenotypic data use can be made directly through EGA, in which the DAC will evaluate each request. The timeframe for final approval is 180 days.

Field-specific reporting

Please select the one below that is the best fit for your research. If you are not sure, read the appropriate sections before making your selection.

☒ Life sciences ☐ Behavioural & social sciences ☐ Ecological, evolutionary & environmental sciences

For a reference copy of the document with all sections, see nature.com/documents/nr-reporting-summary-flat.pdf

Life sciences study design

All studies must disclose on these points even when the disclosure is negative.

Sample size

This is a descriptive study that used the census-based cohort SABE with a sample size analogous to the 1000 Genomes Project. SABE cohort subjects collection followed a two-stage conglomerate sampling method that was used under the probability proportional to size (PPS) criterion. From a total of 1,335 SABE participants enrolled in 2010, samples from 1,200 met quality criteria and were submitted to whole-genome sequencing. Relatedness was assessed by KING, with third-degree relatives as cutoff. We have identifying siblings and duos, keeping one individual at random. The final number of unrelated individuals was 1,171

Data exclusions

Exclusion criteria were pre-established. Up to third degree relatives were excluded. Genotypes below depth of coverage and allele balance values cutoffs were pre-established by CEGH-Filter, as described in Methods.

Replication

We did not perform replication analyses.

Randomization

SABE cohort subjects collection followed a two-stage conglomerate sampling method that was used under the probability proportional to size (PPS) criterion. Apart from initial census-based sampling, all samples that met DNA quality criteria were sequenced.

Blinding

Blinding does not apply to our study design since there was no allocation of cases or controls. On clinical analyses (reclassification of pathogenic variants), investigators that analyzed variant's pathogenicity were unaware of the carriers phenotypes; this information was crossed after reclassification.

Behavioural & social sciences study design

All studies must disclose on these points even when the disclosure is negative.

Study description

Research sample

Sampling strategy

Data collection

Timing

Data exclusions

Non-participation

Randomization

Ecological, evolutionary & environmental sciences study design

All studies must disclose on these points even when the disclosure is negative.

Study description

Research sample

Sampling strategy

Data collection

Timing and spatial scale

Data exclusions

Reproducibility

Randomization

Blinding

Did the study involve field work? ☐ Yes ☐ No

Field work, collection and transport

Field conditions

Location

Access & import/export

Disturbance

Reporting for specific materials, systems and methods

We require information from authors about some types of materials, experimental systems and methods used in many studies. Here, indicate whether each material, system or method listed is relevant to your study. If you are not sure if a list item applies to your research, read the appropriate section before selecting a response.

Materials & experimental systems

n/a	Involved in the study
☒	☐ Antibodies
☒	☐ Eukaryotic cell lines
☒	☐ Palaeontology and archaeology
☒	☐ Animals and other organisms
☐	☒ Human research participants
☒	☐ Clinical data
☒	☐ Dual use research of concern

Methods

n/a	Involved in the study
☒	☐ ChIP-seq
☒	☐ Flow cytometry
☒	☐ MRI-based neuroimaging

Antibodies

Antibodies used

Validation

Eukaryotic cell lines

Policy information about cell lines

Cell line source(s)

Authentication

Mycoplasma contamination

Commonly misidentified lines
(See ICLAC register)

Palaeontology and Archaeology

Specimen provenance

Specimen deposition

Dating methods

☐ Tick this box to confirm that the raw and calibrated dates are available in the paper or in Supplementary Information.

Ethics oversight

Note that full information on the approval of the study protocol must also be provided in the manuscript.

Animals and other organisms

Policy information about studies involving animals; ARRIVE guidelines recommended for reporting animal research

Laboratory animals

Wild animals

Field-collected samples

Ethics oversight

Note that full information on the approval of the study protocol must also be provided in the manuscript.

Human research participants

Policy information about studies involving human research participants

Population characteristics	Census-based cohort from São Paulo city (Brazil) of females and male elderly individuals above 60 years of age that agreed on participating in a follow-up epidemiological study (SABE), with comprehensive phenotypic and biological sampling collections every five years.
Recruitment	Participants are part of a follow-up epidemiological study named SABE and they are recruited based on a probabilistic sampling from the census stratified from 60 years of age and older at the time of collection. Unaccounted biases might include preference of individuals without occupation, or prone to participate in health related studies. General comparisons of SABE participants with other large epidemiological studies do not encounter significant deviations.
Ethics oversight	SABE participants were asked for specific consent on taking part in genomic studies from the year 2010 and beyond. All subjects in the genomic dataset have agreed on participating in this study on written consent forms approved by COEP/CEP/CONEP (Brazilian local and national ethical committee boards) under the following protocols: COEP FSP USP OF.COEP/23/10, CONEP 2044/2014, CEP HIAE 1263-10.

Note that full information on the approval of the study protocol must also be provided in the manuscript.

Clinical data

Policy information about clinical studies

All manuscripts should comply with the ICMJE guidelines for publication of clinical research and a completed CONSORT checklist must be included with all submissions.

Clinical trial registration

Study protocol

Data collection

Outcomes

Dual use research of concern

Policy information about dual use research of concern

Hazards

Could the accidental, deliberate or reckless misuse of agents or technologies generated in the work, or the application of information presented in the manuscript, pose a threat to:

No | Yes

- [ ] [ ] Public health
- [ ] [ ] National security
- [ ] [ ] Crops and/or livestock
- [ ] [ ] Ecosystems
- [ ] [ ] Any other significant area

Experiments of concern

Does the work involve any of these experiments of concern:

No | Yes

- [ ] [ ] Demonstrate how to render a vaccine ineffective
- [ ] [ ] Confer resistance to therapeutically useful antibiotics or antiviral agents
- [ ] [ ] Enhance the virulence of a pathogen or render a nonpathogen virulent
- [ ] [ ] Increase transmissibility of a pathogen
- [ ] [ ] Alter the host range of a pathogen
- [ ] [ ] Enable evasion of diagnostic/detection modalities
- [ ] [ ] Enable the weaponization of a biological agent or toxin
- [ ] [ ] Any other potentially harmful combination of experiments and agents

ChIP-seq

Data deposition

- [ ] Confirm that both raw and final processed data have been deposited in a public database such as GEO.
- [ ] Confirm that you have deposited or provided access to graph files (e.g. BED files) for the called peaks.

Data access links
May remain private before publication.

Files in database submission

Genome browser session
(e.g. UCSC)

Methodology

Replicates

Sequencing depth

Antibodies

Peak calling parameters

Data quality

Software

Flow Cytometry

Plots

Confirm that:

- [ ] The axis labels state the marker and fluorochrome used (e.g. CD4-FITC).
- [ ] The axis scales are clearly visible. Include numbers along axes only for bottom left plot of group (a 'group' is an analysis of identical markers).
- [ ] All plots are contour plots with outliers or pseudocolor plots.
- [ ] A numerical value for number of cells or percentage (with statistics) is provided.

Methodology

Sample preparation

Instrument

Software

Cell population abundance

Gating strategy

☐ Tick this box to confirm that a figure exemplifying the gating strategy is provided in the Supplementary Information.

Magnetic resonance imaging

Experimental design

Design type

Design specifications

Behavioral performance measures

Acquisition

Imaging type(s)

Field strength

Sequence & imaging parameters

Area of acquisition

Diffusion MRI ☐ Used ☐ Not used

Preprocessing

Preprocessing software

Normalization

Normalization template

Noise and artifact removal

Volume censoring

Statistical modeling & inference

Model type and settings

Effect(s) tested

Specify type of analysis: ☐ Whole brain ☐ ROI-based ☐ Both

Statistic type for inference
(See Eklund et al. 2016)

Correction

Models & analysis

n/a	Involved in the study
☐	☐ Functional and/or effective connectivity
☐	☐ Graph analysis
☐	☐ Multivariate modeling or predictive analysis

Functional and/or effective connectivity

Graph analysis

Multivariate modeling and predictive analysis
